# OMO-1 reduces progression and enhances cisplatin efficacy in a 4T1-based non-c-MET addicted intraductal mouse model for triple-negative breast cancer

**DOI:** 10.1038/s41523-021-00234-8

**Published:** 2021-03-17

**Authors:** Jonas Steenbrugge, Niels Vander Elst, Kristel Demeyere, Olivier De Wever, Niek N. Sanders, Wim Van Den Broeck, Eric Ciamporcero, Timothy Perera, Evelyne Meyer

**Affiliations:** 1 Laboratory of Biochemistry, Department of Pharmacology, Toxicology and Biochemistry, Faculty of Veterinary Medicine, Ghent University, Merelbeke, Belgium; 2 Cancer Research Institute Ghent (CRIG), Ghent, Belgium; 3 Laboratory of Experimental Cancer Research, Department of Human Structure and Repair, Ghent University, Ghent, Belgium; 4 Laboratory of Gene Therapy, Department of Nutrition, Genetics and Ethology, Faculty of Veterinary Medicine, Ghent University, Merelbeke, Belgium; 5 Department of Morphology, Faculty of Veterinary Medicine, Ghent University, Merelbeke, Belgium; 6 OCTIMET Oncology NV, Beerse, Belgium

**Keywords:** Targeted therapies, Cancer models, Cancer microenvironment, Breast cancer

## Abstract

c-MET is considered a driver of cancer progression, impacting tumor growth and tumor-supporting stroma. Here, we investigated the therapeutic efficacy of OMO-1, a potent and selective c-MET inhibitor, in an immunocompetent intraductal mouse model for triple-negative breast cancer (TNBC). OMO-1 reduced non-c-MET addicted 4T1 tumor progression dose dependently as monotherapeutic and provided additional disease reduction in combination with cisplatin. At the stromal level, OMO-1 significantly reduced neutrophil infiltration in 4T1 tumors, promoted immune activation, and enhanced cisplatin-mediated reduction of tumor-associated macrophages. OMO-1 treatment also reduced 4T1 tumor hypoxia and increased expression of pericyte markers, indicative for vascular maturation. Corroborating this finding, cisplatin delivery to the 4T1 primary tumor was enhanced upon OMO-1 treatment, increasing cisplatin DNA-adduct levels and tumor cell death. Although verification in additional cell lines is warranted, our findings provide initial evidence that TNBC patients may benefit from OMO-1 treatment, even in cases of non-c-MET addicted tumors.

## Introduction

The c-MET pathway is known to drive cellular malignant processes and has been reported as a target for anticancer therapy^1–4^. Bound by hepatocyte growth factor (HGF), the receptor tyrosine kinase c-MET provides necessary survival and migration signals during embryogenesis^2–4^. Whilst mutations in this pathway stimulate the growth and metastasis of some tumors, it also plays a major role in the tumor stroma. Indeed, HGF/c-MET signaling stimulates the recruitment of neutrophils^5^ and can induce the production of anti-inflammatory cytokines^6^. Moreover, there have been clear indications that c-MET is involved in neovascularization and blood vessel homeostasis^6^. In the context of triple-negative breast cancer (TNBC), c-MET is overexpressed in about 52% of TNBC patient tumors, and high c-MET expression is correlated with worse disease-free as well as overall survival^7,8^. These findings have prompted the clinical testing of c-MET signaling blockers for TNBC, including ATP-competitive small molecule inhibitors^1^. Crizotinib and cabozantinib, for example, showed promising results in TNBC preclinical models and patients^9–12^. A recently developed highly selective ATP-competitive c-MET inhibitor OMO-1 (formerly referred to as JNJ-38877618, chemical structure: 6-(difluoro(6-(pyridin-4-yl)^1,2,4^triazolo[4,3-b]pyridazin-3-yl)methyl)quinoline) has also raised attention as an alternative agent^1,13^. More specifically, preclinical studies with OMO-1 showed its superior inhibitory activity against wild-type c-MET compared to crizotinib (i.e., 2 versus 11.7 nM IC_50_)^13^. A phase I/II clinical trial (NCT03138083) evaluated the safety, tolerability, and preliminary therapeutic efficacy of OMO-1 in c-MET pathway-driven solid tumors^13,14^. Nevertheless, the efficacy of OMO-1 in TNBC has not yet been investigated preclinically.

Here, we evaluated the efficacy of OMO-1 in an innovative 4T1-based immunocompetent intraductal mouse model for TNBC. In contrast to the classical fat pad model, the intraductal model more closely resembles the human disease process as intraductally inoculated tumors grow from within the mammary ducts and undergo ductal breakthrough to invade the mammary fat pad and metastasize to distant organs^15–18^. The use of immunocompetent mice allows to study the possible impact of OMO-1 on tumor immunology. Moreover, as 4T1 tumors are highly hypoxic and angiogenic^16,18^, the use of a 4T1-based model allows the elucidation of a potential impact of OMO-1 on intratumoral hypoxia and tumor vasculature.

## Results

### OMO-1 decreases non-c-MET addicted tumor progression and has additional therapeutic effects in combination with cisplatin

**Fig. 1:**
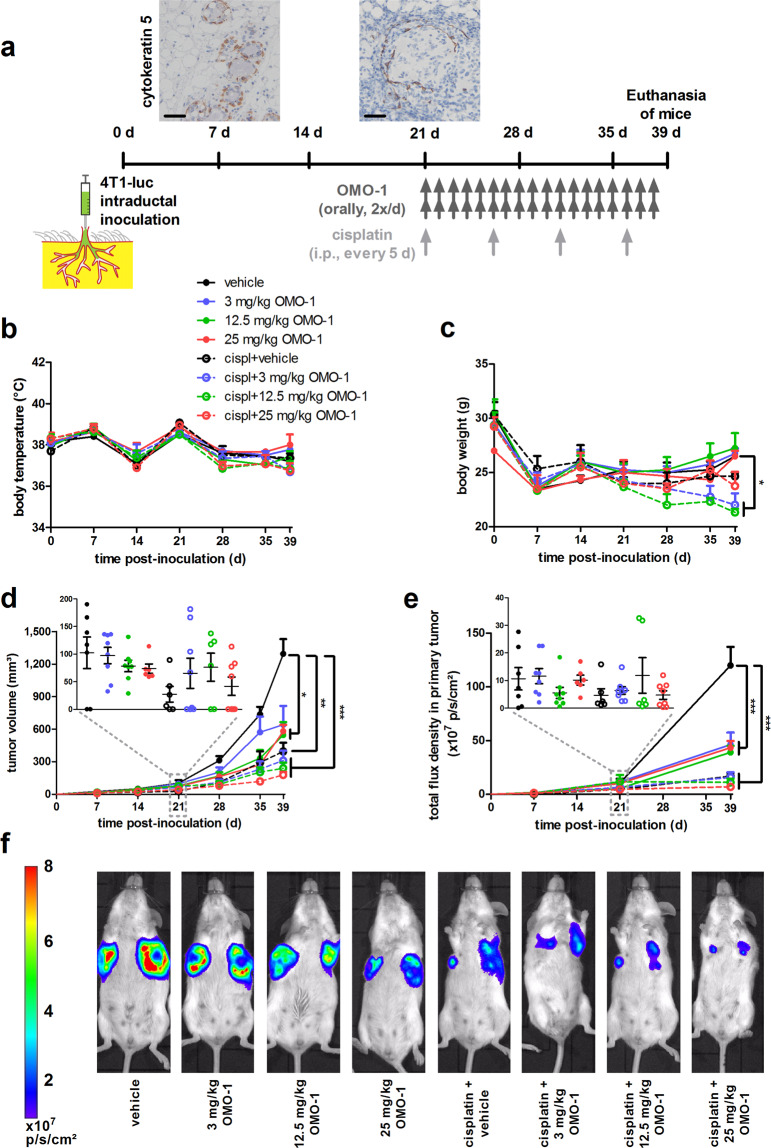
OMO-1 treatment decreases primary tumor growth in a 4T1-based intraductal model. **a** Experimental timeline of the study, including immunohistochemistry for the myoepithelial cell marker cytokeratin 5 on primary tumor paraffin sections at 7 and 21 days p.i. Scale bar = 50 µm. Mouse body temperature (**b**) and weight (**c**) measurements (of the 29 included tumor-bearing mice, 4 mice were treated with vehicle, 4 mice with 3 mg/kg OMO-1, 4 mice with 12.5 mg/kg OMO-1, 3 mice with 25 mg/kg OMO-1, 3 mice with cisplatin + vehicle, 4 mice with cisplatin + 3 mg/kg OMO-1, 3 mice with cisplatin + 12.5 mg/kg, and 4 mice with cisplatin + 25 mg/kg OMO-1). **d** Primary tumor growth based on tumor volume measurements (*n* = 7 for vehicle, *n* = 6 for 25 mg/kg OMO-1, cisplatin + vehicle and cisplatin + 12.5 mg/kg OMO-1, *n* = 8 for all other treatment groups at 39 days p.i.). Insert shows statistically nonsignificant differences in primary tumor volumes between the randomized treatment groups at the start of treatment (i.e., 21 days p.i.). **e** Primary tumor growth based on in vivo imaging (measured as total flux density (p/s/cm^2^)) (*n* = same as for tumor volume measurements, except *n* = 4 for 25 mg/kg OMO-1 at 39 days p.i.). Insert shows statistically nonsignificant differences in total flux density in primary tumors between the randomized treatment groups at the start of treatment (i.e., 21 days p.i.). **f** Representative images of the in vivo bioluminescence signals at 39 days p.i. in each treatment group. Data in **b**–**e** are presented as the means ± standard error of the mean (SEM). *P < 0.05, **P < 0.01, ***P < 0.001.

Following intraductal inoculation of luciferase-expressing 4T1 tumor cells through the teat canal of syngeneic and lactating BALB/c mice, primary tumors were allowed to grow for 21 days. Based on previous studies by our group^15–18^ and cytokeratin 5 stainings for myoepithelial cells, 4T1 tumor cells remained inside the mammary ducts surrounded by an intact epithelial barrier until 7 days post inoculation (p.i.), characteristic for the ductal carcinoma in situ (DCIS) stage (Fig. 1a). By 21 days p.i., primary tumors progressed to invasive carcinoma, characterized by tumor cells that were breaking through the ductal epithelial barrier as shown by cytokeratin 5 stainings, in order to invade the mammary fat pad (Fig. 1a). Mice were dosed either with OMO-1 (i.e., OMO-1 monotherapy) or with the OMO-1 vehicle as control, both administered orally 2×/day (bis in die, b.i.d.) for a period of 18 days. To investigate the influence of OMO-1 on chemotherapy, cisplatin was administered every 5 days intraperitoneally (i.p.) for a period of 18 days to a group of tumor-bearing mice in combination with b.i.d. oral administration of either OMO-1 (i.e., cisplatin + OMO-1) or the OMO-1 vehicle (i.e., cisplatin + vehicle) as control. Treatment with OMO-1 did not alter body temperature, either as monotherapy or in combination with cisplatin (Fig. 1b). OMO-1 monotherapy also did not alter body weight (Fig. 1c). On the other hand, treatment with cisplatin with or without OMO-1 decreased body weight at the endpoint (i.e., 39 days p.i.) compared to vehicle (Fig. 1c), but only the decrease in the cisplatin + 3 mg/kg and 12.5 mg/kg OMO-1 combination treatment groups was statistically significant. Of note, all mice showed weight loss in the first week p.i. due to decreased milk production after weaning of the pups. At the start of treatment (i.e., 21 days p.i.), baseline tumor growth did not statistically differ between the randomized treatment groups based on tumor volume measurements as well as on in vivo imaging. Yet, both techniques identified a significant and dose-dependent reduction in primary tumor progression with OMO-1 monotherapy at 39 days p.i., (i.e., following 18 days of treatment) (Fig. 1d–f). Although the cisplatin and OMO-1 combination-treated tumors were smaller than cisplatin + vehicle-treated tumors, this reduction did not reach significance possibly because of the small group sizes. Ki67 stainings and index calculations identified a significant decrease in cellular proliferation of primary tumors treated with 12.5 and 25 mg/kg OMO-1 monotherapy compared to vehicle treatment. This decrease was more pronounced both with cisplatin + vehicle and cisplatin + OMO-1 combination treatment (Supplementary Fig. 1a). Importantly, cisplatin + 25 mg/kg OMO-1 combination treatment significantly reduced the Ki67 staining compared to cisplatin + vehicle treatment (Supplementary Fig. 1a). To identify whether c-MET addiction of 4T1 tumor cells and their concomitant sensitivity to c-MET blockade could explain the reduction in cell proliferation upon OMO-1 treatment, we evaluated c-MET expression in 4T1 primary tumors. Yet, the expression of c-MET by western blot analysis was limited, based on two independent antibodies on the same blot, showing only a very faint (nonspecific) band between 130 and 180 kDa or between 100 and 130 kDa (expected molecular weights are 145 kDa for the mature c-MET β-subunit and 170 kDa for the pro-c-MET) in both treated and untreated primary tumor samples (Supplementary Fig. 1b). Corroborating the ex vivo western blotting, 4T1 cells showed limited c-MET expression in vitro (Supplementary Fig. 1b). Western blotting on lysates of cultured 4T1 cells following 48 h treatment with either OMO-1 or dimethyl sulfoxide (DMSO, i.e., OMO-1 vehicle solution) in combination with or without cisplatin showed similar phosphorylated Akt and p44/42 MAPK (Erk1/2) levels in all in vitro treatment conditions (Supplementary Fig. 2a). In contrast, the c-MET amplified human non-small cell lung carcinoma cell line NCI-H441 serving as positive control showed strong and dose-dependent reduction in phosphorylated c-MET levels as well as in phosphorylated downstream effectors Gab2, Akt, MEK1/2, and p42/44 MAPK (Erk1/2) upon OMO-1 treatment (Supplementary Fig. 2b). In vitro 4T1 tumor cell growth was also unaffected by 48 h treatment with OMO-1 compared to DMSO control as determined by neutral red uptake assay, which is to be expected from a non-c-MET addicted cell line (Supplementary Fig. 3a). Only cisplatin treatment significantly inhibited in vitro cellular growth and OMO-1 did not have an additive treatment effect. Ki67 immunocytochemical stainings and the proliferation index of the treated 4T1 tumor cell cultures confirmed these findings (Supplementary Fig. 3b). Immunocytochemical stainings for cleaved caspase 3 showed that the decreased in vitro cellular growth with cisplatin treatment was associated with an increase in cell death (Supplementary Fig. 3b).

Analysis of 4T1 bioluminescence in isolated axillary lymph nodes and lungs of the tumor-bearing mice identified a significant reduction in metastases with cisplatin, either in combination with or without OMO-1, compared to vehicle treatment (Supplementary Fig. 4a). Of note, the intensity of bioluminescent signals was higher in lungs compared to axillary lymph nodes in vehicle-treated mice, which verifies the lungs as primary metastatic site for 4T1 cells. Hematoxylin and eosin (H&E) histology (Supplementary Fig. 4b) and Ki67 staining (Supplementary Fig. 5) identified infiltrating and proliferative tumor cells based on morphology and showed that the cisplatin + 25 mg/kg OMO-1 combination treatment induced complete elimination of the metastases in axillary lymph nodes and lungs compared to cisplatin + vehicle treatment. Bioluminescent signals were also detected in the liver of tumor-bearing mice and were significantly reduced with cisplatin treatment (Supplementary Fig. 4a). H&E histology showed cellular infiltration from the vasculature into the liver tissue (Supplementary Fig. 4b) and Ki67 stainings further verified the presence of proliferative cells, which significantly reduced with OMO-1 compared to vehicle treatment either in combination with or without cisplatin (Supplementary Fig. 6). Interestingly, the Ki67^+^ proliferating cells were not identified as tumor cells based on their negative pan-cytokeratin staining, but as myeloid cells based on their positive CD11b staining (Supplementary Fig. 6). Quantification of the CD11b stainings in liver tissue of the different treatment groups showed a significant reduction in myeloid cell content with OMO-1 treatment in combination with or without cisplatin (Supplementary Fig. 6), indicative for a decrease in disease progression and myeloid cell-mediated immunosuppression. Of note, the observed bioluminescent signals in the liver are likely not derived from the proliferative myeloid cells, but rather background from 4T1 cells in blood that excessively flows through and can be contained in the liver.

### OMO-1 decreases neutrophil infiltration, induces immune stimulation, and in combination with cisplatin reduces tumor-associated macrophages (TAMs)

Splenomegaly has been associated with 4T1 tumor progression and leukemoid reactions^15,16,18–21^. Based on spleen weights, tumor-bearing mice receiving vehicle suffered from severe splenomegaly (Supplementary Fig. 7a). Cisplatin either with or without OMO-1 significantly reduced splenomegaly compared to vehicle treatment, i.e., to near healthy spleen weights, but OMO-1 showed no additional effect compared to cisplatin + vehicle treatment (Supplementary Fig. 7a).

Chitinase 3-like 1 (CHI3L1) and lipocalin 2 (LCN2) have been utilized as immune-related biomarkers for disease progression in breast cancer patients^22–24^ and in the 4T1-based immunocompetent intraductal mouse model for TNBC^15,16,18^. Similar to the splenomegaly, CHI3L1 and LCN2 levels were reduced in primary tumors, sera, and spleens upon cisplatin treatment either with or without OMO-1 compared to vehicle treatment (Supplementary Fig. 7b–d). Again, OMO-1 showed no additional effect compared to cisplatin + vehicle.

The levels of a panel consisting of 11 selected cytokines were subsequently measured in primary tumor lysates to provide a broader view of the local immune responses in the treated mice. In primary tumors, OMO-1 monotherapy significantly reduced the levels of macrophage inflammatory protein (MIP)-2, a major neutrophil chemoattractant, compared to vehicle treatment (Supplementary Fig. 8a). Interestingly, primary tumor MIP-2 levels also significantly decreased with cisplatin + OMO-1 combination but not with cisplatin + vehicle treatment, both compared to vehicle treatment (Supplementary Fig. 8a). Cisplatin treatment had a profound immunological effect by significantly reducing the levels of the primary tumor cytokines B-cell activating factor (BAFF) and interleukin (IL)-1β compared to vehicle treatment (Supplementary Fig. 8a). The levels of eight other cytokines, i.e., granulocyte colony-stimulating factor (G-CSF), interferon (IFN)-γ, IL-4, IL-6, IL-10, monocyte chemoattractant protein (MCP)-1, tumor necrosis factor (TNF)-α, and transforming growth factor (TGF)-β1, showed no statistically significant changes with either of the treatments.

Protein array analysis highlighted 11 additional protein markers that were significantly altered in primary tumor lysates upon OMO-1 monotherapy and, in some cases, also upon cisplatin + OMO-1 combination therapy (Supplementary Fig. 8b). Immune-associated proteins including CD40, C-X3-C motif chemokine ligand 1, IL-1ra, IL-12 p40, and regenerating family member 3 gamma showed significantly increased levels in the 25 mg/kg OMO-1-treated compared to vehicle treatment group, indicating that OMO-1 induced stimulation of the immune system (Supplementary Fig. 8c). OMO-1 further induced a tumor suppressive effect based on enhanced osteoprotegerin and Wnt1-inducible signaling pathway protein 1 (WISP-1) levels (Supplementary Fig. 8c). WISP-1 was not OMO-1-specific as cisplatin + vehicle treatment induced a similar enhancement. The four remaining proteins are linked to vascular stability and integrity, which impacts tumor escape and immune infiltration. More specifically, treatment with OMO-1 significantly increased the production of endoglin, fibroblast growth factor (FGF)-1, intercellular adhesion molecule (ICAM)-1, and matrix metalloproteinase (MMP)-3 compared to vehicle treatment (Supplementary Fig. 8c). In combination with cisplatin, OMO-1 induced a significant increase in endoglin and FGF-1 compared to vehicle (Supplementary Fig. 8c).

**Fig. 2:**
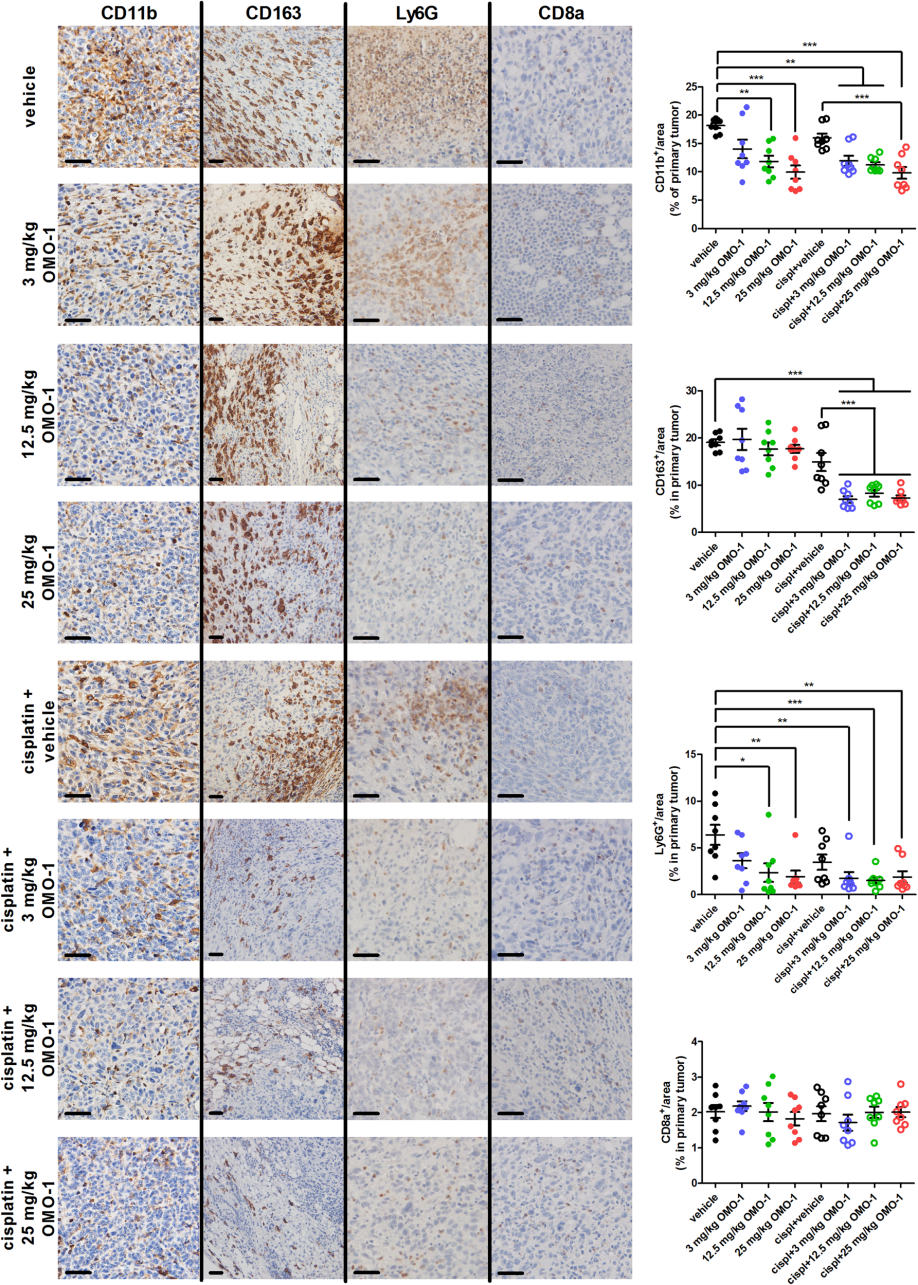
OMO-1 impacts tumor infiltrating neutrophils and immunosuppressive macrophages in a 4T1-based intraductal model. Immunohistochemistry for CD11b (pan-myeloid cells), CD163 (tumor-associated macrophages), Ly6G (neutrophils), and CD8a (cytotoxic T-cells) on paraffin sections of primary tumors from the different treatment groups at 39 days p.i. (*n* = 8; 2 slides per treatment group for each marker with 4 images per slide). Scale bars = 50 µm. Data are presented as the means ± SEM. *P < 0.05, **P < 0.01, ***P < 0.001.

Immunohistochemical staining allowed further visualization of the immune components of the TME in the different treatment groups. OMO-1 in combination with or without cisplatin significantly reduced myeloid cell numbers in primary tumors compared to vehicle treatment based on CD11b stainings (Fig. 2). Expression of the CD163 TAM marker significantly decreased in primary tumors treated with cisplatin + OMO-1 combination treatment, when compared to vehicle and cisplatin + vehicle treatment (Fig. 2). Expression of the Ly6G tumor-associated neutrophil (TAN) marker significantly decreased in primary tumors treated with 12.5 and 25 mg/kg OMO-1 monotherapy and in the cisplatin + OMO-1 combination groups, but was not observed with cisplatin + vehicle treatment when compared to vehicle treatment (Fig. 2). This observation corroborates the MIP-2 levels observed upon cytokine profiling in primary tumors (Supplementary Fig. 8a). Expression of the CD8a cytotoxic T-cell marker did not significantly differ between the treatment groups (Fig. 2). Similarly, expression of the granzyme B T-cell activity marker and immune checkpoint proteins programmed death (PD)-1 and cytotoxic T-lymphocyte antigen (CTLA)-4 did not significantly decrease with either treatment based on immunohistochemistry (Supplementary Fig. 9a) or on protein levels in tumor lysates (Supplementary Fig. 9b). These observations corroborate the unchanged T-cell-associated IFN-γ and TNF-α primary tumor levels with either treatment based on cytokine profiling (Supplementary Fig. 8a).

### OMO-1 decreases hypoxia and is associated with increased pericyte markers in primary tumors

**Fig. 3:**
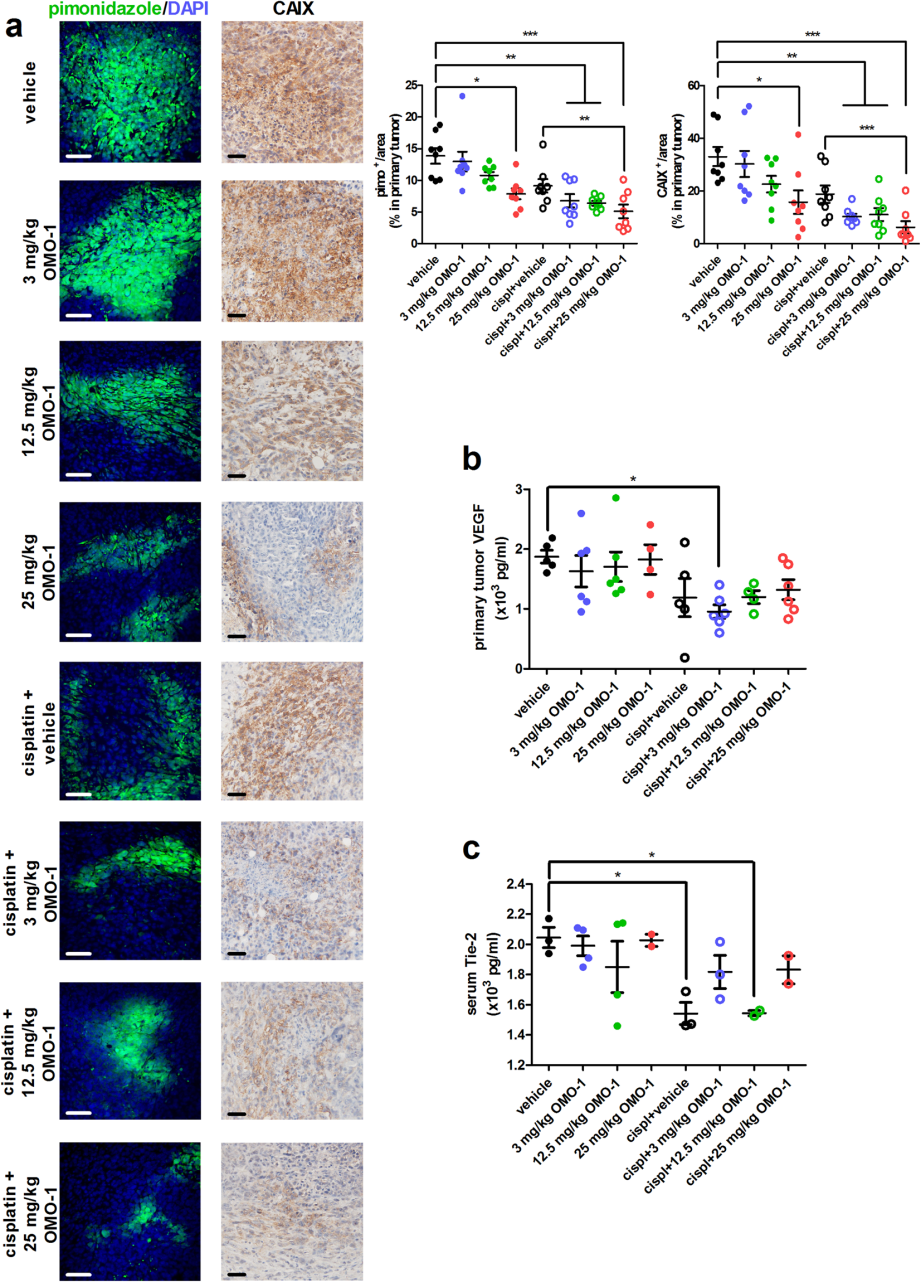
OMO-1 treatment reduces tumor hypoxia without affecting vascular formation in a 4T1-based intraductal model. **a** Immunohistochemistry for the hypoxia markers pimonidazole and CAIX on paraffin sections of primary tumors from the different treatment groups at 39 days p.i. (*n* = 8; 2 slides per treatment group for each marker with 4 images per slide). Pimonidazole adducts are identified through FITC fluorescence and DAPI identifies cellular nuclei through blue fluorescence. Scale bars = 50 µm. **b** VEGF levels in primary tumors from the different treatment groups at 39 days p.i. (*n* = 5 for vehicle and cisplatin + vehicle, *n* = 4 for 25 mg/kg OMO-1 and cisplatin + 12.5 mg/kg OMO-1, *n* = 6 for all other treatment groups). **c** Tie-2 levels in serum from the different treatment groups at 39 days p.i. (*n* = 3 for vehicle, cisplatin + vehicle and cisplatin + 3 mg/kg OMO-1, *n* = 2 for 25 mg/kg OMO-1, cisplatin + 12.5 mg/kg OMO-1, and cisplatin + 25 mg/kg OMO-1, *n* = 4 for all other treatment groups). Data are presented as the means ± SEM. *P < 0.05, **P < 0.01, ***P < 0.001.

4T1 tumor cells have been shown to grow aggressively and form highly hypoxic primary tumors in the immunocompetent intraductal mouse model for TNBC^16,18^. To investigate whether OMO-1 treatment impacts 4T1 tumor hypoxia concomitant with the reduced primary tumor growth, pimonidazole hydrochloride was administered before sacrifice. Pimonidazole is a 2-nitroimidazole-based agent that forms adducts with thiol groups in hypoxic cells at oxygen levels below 10 mmHg^25,26^. An FITC-conjugated antibody that specifically recognizes pimonidazole adducts allows to visualize poorly oxygenated regions in histological sections of primary tumors. The amount of pimonidazole adduct staining has been described as proportional to the level of hypoxia in primary tumors^26^. Detectable levels of pimonidazole adducts significantly decreased upon monotherapy with 25 mg/kg OMO-1 compared to vehicle treatment (Fig. 3). Moreover, when combined with cisplatin, OMO-1 established a significant reduction in pimonidazole adduct staining compared to cisplatin + vehicle treatment. Staining for carbonic anhydrase 9 (CAIX), a hypoxia-associated enzyme^27,28^, corroborated these findings (Fig. 3). Both pimonidazole and CAIX stainings identified that cisplatin + vehicle treatment did not significantly decrease tumor hypoxia compared to vehicle treatment, suggesting that the effects of OMO-1 correlate with its activity on the tumor vasculature and oxygenation.

**Fig. 4:**
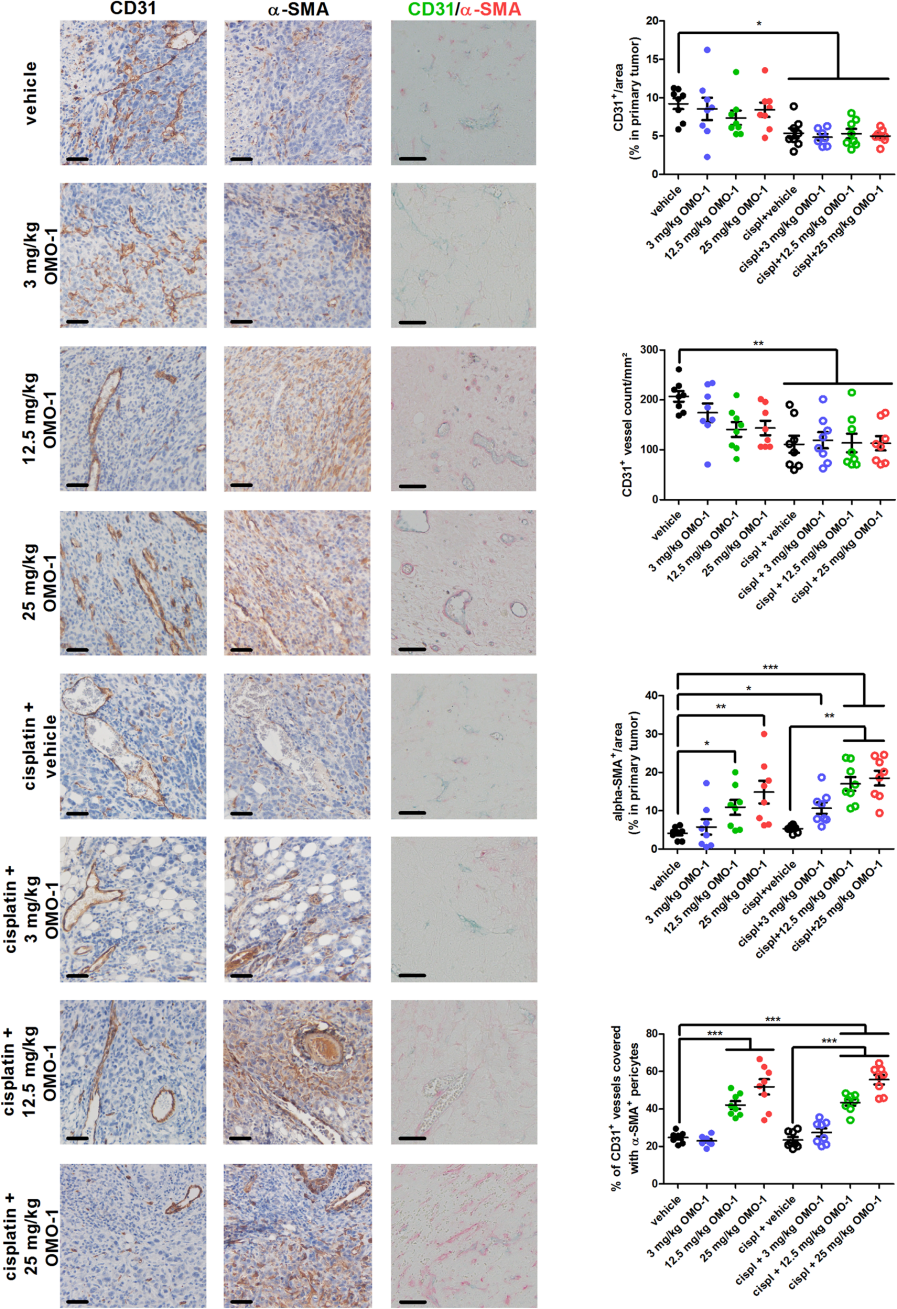
OMO-1 treatment increases vascular pericytes in primary tumors of a 4T1-based intraductal model. Immunohistochemistry for the endothelial cell marker CD31 and the pericyte marker α-SMA and double staining immunohistochemistry for CD31 and α-SMA on paraffin sections of primary tumors from the different treatment groups at 39 days p.i. (*n* = 8; 2 slides per treatment group for each marker with 4 images per slide). CD31^+^ vessels were manually counted on 20× magnified microscopic images. The proportion of α-SMA-covered CD31^+^ vessels among total counted CD31^+^ vessels was also determined on 20× magnified microscopic images. Scale bars = 50 µm. Data are presented as the means ± SEM. *P < 0.05, **P < 0.01, ***P < 0.001.

Interestingly, OMO-1 monotherapy did not alter primary tumor levels of the angiogenesis biomarker vascular endothelial growth factor (VEGF). Only cisplatin treatment in combination with 3 mg/kg OMO-1 significantly decreased the primary tumor VEGF levels compared to vehicle treatment (Fig. 3b). Serum levels of Tie-2, a biomarker for VEGF inhibition^29^, stainings for the endothelial cell marker CD31 and CD31^+^ vessel counts on primary tumor sections corroborated these VEGF results with significant reduction upon cisplatin treatment (Figs. 3c and 4). Of note, whereas CD31^+^ stainings and vessel counts decreased in all cisplatin-treated groups, serum Tie-2 levels only significantly decreased in the cisplatin + vehicle and cisplatin + 12.5 mg/kg OMO-1 group. Overall, these results identify that OMO-1 does not alter hypoxia via the modulation of vascular factors and vascular formation in 4T1 primary tumors.

The reduction in tumor hypoxia by OMO-1 may alternatively be explained by increased pericyte coverage of tumor vasculature, an indicator of blood vessel maturation, resulting in increased tumor oxygenation and vessel perfusion^30,31^. To this end, positive staining for c-MET in the vascular bed was first verified on primary tumor sections (Supplementary Fig. 10a). OMO-1 treatment did not influence the vascular expression of c-MET in primary tumors based on qualitative assessment of this immunohistochemical staining (Supplementary Fig. 10a). In line with a potential OMO-1-mediated vessel normalization, stainings for α-smooth muscle actin (SMA) (Fig. 4) and platelet-derived growth factor receptor (PDGFR)-β (Supplementary Fig. 10b), two established pericyte markers^27,28^, dose dependently increased in primary tumors upon OMO-1 monotherapy and cisplatin + OMO-1 combination treatment compared to vehicle treatment. Double staining immunohistochemistry for colocalization of CD31 and α-SMA and subsequent manual counting confirmed the dose-dependent increase in vascular pericytes upon OMO-1 treatment (Fig. 4).

Pericyte coverage is intricately linked to the family of angiopoietins (Ang), with Ang-1 and Ang-2 being the most influential factors^30,31^. Ang-1 is reported to increase pericyte survival through activation of Tie-2 expression on endothelial cells as well as pericytes^30–32^. Ang-2 on the other hand is reported to counteract the vascular stabilization effect of Ang-1 by competitively binding to Tie-2^30–32^. As we hypothesized that the increased pericyte abundance in primary tumors may be explained by an imbalance between Ang-1 and -2, the primary tumor levels of both Ang family members were determined. Stainings for Ang-1 in primary tumors significantly increased upon treatment with 12.5 and 25 mg/kg OMO-1 compared to vehicle (Supplementary Fig. 10b). Cisplatin + 12.5 mg/kg OMO-1- and cisplatin + 25 mg/kg OMO-1-treated mice also showed increased Ang-1 positivity in primary tumors compared to cisplatin + vehicle-treated mice (Supplementary Fig. 10b). In contrast, primary tumor levels of Ang-2 were not affected by either treatment, based on western blot and enzyme-linked immunosorbent assay (ELISA) measurements (Supplementary Fig. 11a, b). In addition, primary tumor Tie-2 levels were measured through ELISA and were also found to be similar in all treatment groups (Supplementary Fig. 11c).

As tumor cells have been, in certain cases, reported to resemble pericytes through a process referred to as epithelial-to-pericyte transition (EPT)^33,34^, it was investigated whether 4T1 tumor cells were able to express any pericyte markers upon OMO-1 treatment. Yet, western blotting with lysates from in vitro cultured and 48-h-treated 4T1 cells did not detect expression of α-SMA and PDGFR-β (Supplementary Fig. 11d).

### OMO-1 increases cisplatin-induced cell death through enhanced drug delivery

**Fig. 5:**
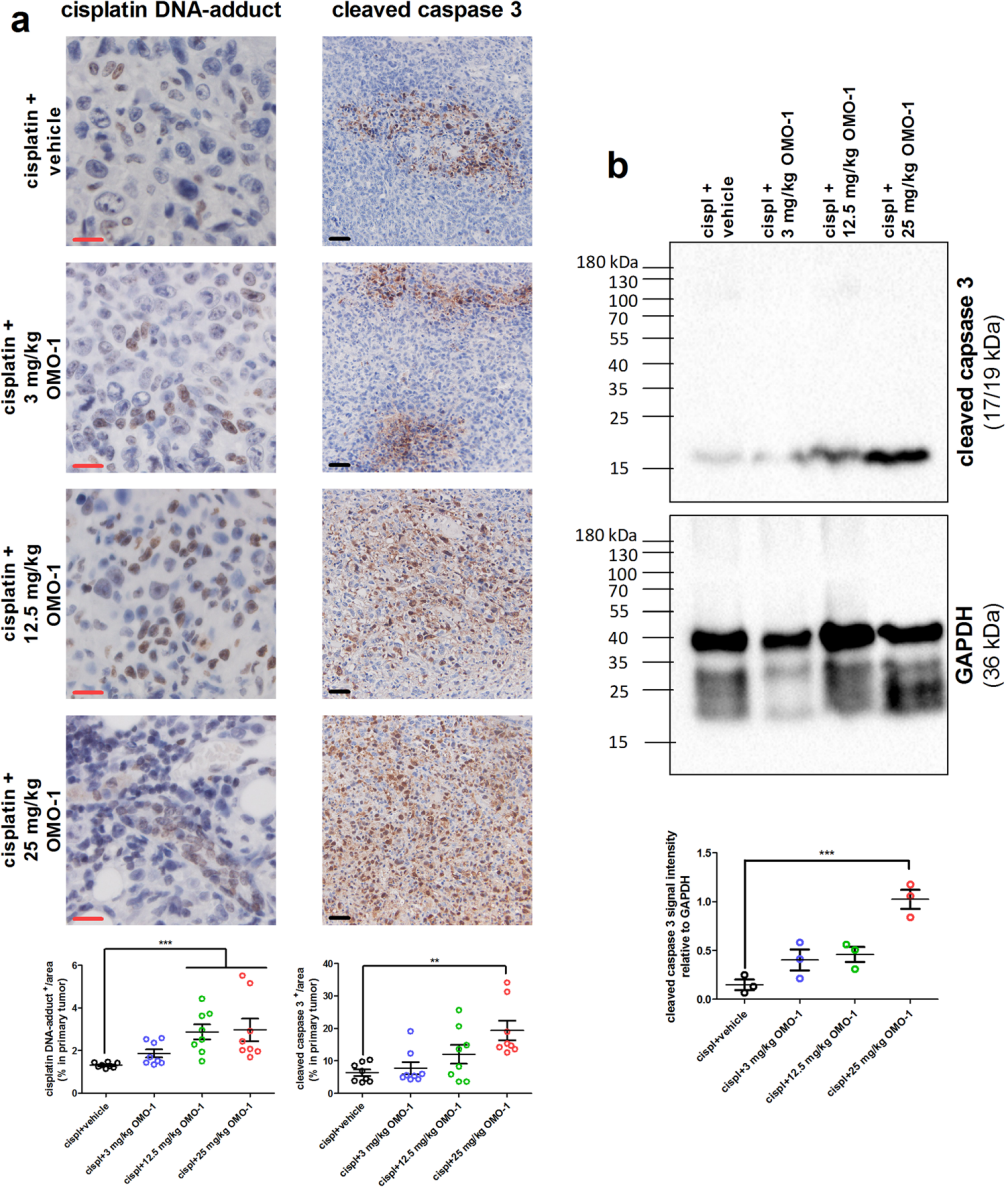
OMO-1 treatment increases cisplatin-mediated cell death in primary tumors of a 4T1-based intraductal model. **a** Immunohistochemistry for cisplatin DNA-adducts and the cell death marker cleaved caspase 3 on paraffin sections of primary tumors at 39 days p.i. treated with cisplatin + vehicle and cisplatin + OMO-1 (*n* = 8; 2 slides per treatment group for each marker with 4 images per slide). Black scale bars = 50 µm, red scale bars = 20 µm. **b** Western blot for cleaved caspase 3 levels and GAPDH control levels in lysates of primary tumors at 39 days p.i. treated with cisplatin + vehicle and cisplatin + OMO-1 (*n* = 3 for each treatment group). Data are presented as the means ± SEM. **P < 0.01, ***P < 0.001.

Based on the potential increase in pericyte coverage upon OMO-1 treatment, as suggested by the selective increase in pericyte markers, we further hypothesized that the delivery of cisplatin to primary tumors could also be increased. Cisplatin DNA-adduct positivity in primary tumors increased upon OMO-1 treatment in a dose-dependent manner, being significantly higher with cisplatin + 12.5 mg/kg OMO-1 and cisplatin + 25 mg/kg OMO-1 combination treatment compared to cisplatin + vehicle treatment (Fig. 5a). As this increased cisplatin delivery would result in increased cell death within the primary tumors, the levels of the active apoptotic primary tumor biomarker cleaved caspase 3 were determined. Western blotting and immunohistochemistry both demonstrated an increase of cleaved caspase 3 levels in primary tumor lysates, which was OMO-1 dose dependent (Fig. 5a, b). More specifically, cleaved caspase 3 levels were significantly higher in cisplatin + 25 mg/kg OMO-1-treated primary tumors compared to cisplatin + vehicle-treated primary tumors.

## Discussion

One strategy to tackle cancer is by inhibiting pathways that increase tumor cell survival, proliferation, and migration such as the HGF/c-MET pathway, which is currently being investigated in multiple cancer types^1–4^. Although c-MET inhibition as anticancer strategy has provided promising preliminary results, there is still a need for both more effective and selective c-MET inhibitors which could then be combined with other treatment regimens such as chemotherapy or immunotherapy^7,35^. In the current proof-of-concept study, the novel, potent, and selective c-MET inhibitor OMO-1 was evaluated in the context of TNBC using a validated 4T1-based immunocompetent intraductal mouse model.

Oral treatment with OMO-1 was well tolerated, but body weights decreased upon cisplatin treatment either alone or in combination with OMO-1. Cisplatin chemotherapy is known to cause gastro-intestinal problems which ultimately result in decreased body weight^36^. Although cisplatin + 3 mg/kg OMO-1 and cisplatin + 12.5 mg/kg OMO-1 combination treatment resulted in the lowest, yet significant, decrease in body weight compared to vehicle and OMO-1 monotherapy, it is unlikely that OMO-1 induced a higher toxicity in combination with cisplatin. Indeed, mice treated with cisplatin in combination with the highest dose of OMO-1 had a similar body weight compared to mice treated with cisplatin + vehicle.

OMO-1 monotherapy induced a significant decrease in tumor progression and proliferation at the invasive edge, providing an additional treatment effect when combined with cisplatin, which strongly indicates a potential benefit for combination therapy in a clinical setting. Metastases were verified in axillary lymph nodes and lungs with OMO-1 treatment again providing an added metastatic reduction in combination with cisplatin. Liver tissue did not contain metastases, but showed an abundance of proliferative myeloid cells, which decreased upon treatment with OMO-1. Indeed, this is likely an indication for 4T1-stimulated extramedullary hematopoiesis in the liver and is known to drive disease progression and immunosuppressive activity^37,38^. Although in contrast to previous reports^39,40^, very low c-MET expression was detectable in in vitro 4T1 cell lysates as well as in vivo primary tumor lysates on western blot (and immunohistochemistry). In line with this observation, OMO-1-mediated c-MET inhibition also did not affect phosphorylation of known downstream c-MET effectors Akt and p44/42 MAPK (Erk1/2) in 4T1 cell lysates nor 4T1 cell growth in vitro. Consequently, as the progression of the 4T1 cell line used in our study is not addicted to c-MET signaling, our data suggest that OMO-1 does not reduce in vivo 4T1 tumor progression solely by targeting the mammary tumor cells.

At the immunological level, OMO-1 had an inhibitory effect on neutrophil infiltration in primary tumors. Such TAN blockade by c-MET inhibitors has been proposed to be detrimental because of the antitumoral properties attributed to TANs^5^. However, it has also been reported that TANs can be immunosuppressive—albeit in response to immunotherapy—and that immunotherapeutic efficacy is improved when neutrophil recruitment into tumors is impaired by c-MET inhibition^41^. Other studies have also highlighted a critical role for neutrophils in stimulating (breast) cancer progression^42–45^. In line with the hypothesis of a more immune activated TME, OMO-1 treatment induced enhanced immunostimulatory and tumor suppressive markers in primary tumor lysates. Moreover, in combination with cisplatin, OMO-1 provided a significant reduction in TAM-mediated immunosuppression.

The reduction in tumor hypoxia is potentially an additional antitumor activity of OMO-1. Indeed, hypoxia has been associated with poor prognosis and known to drive metastases^46^. Our data show that the OMO-1-mediated hypoxia reduction was not only due to a decrease in primary tumor growth but rather to a direct effect on the tumor vasculature. c-MET and its ligand HGF are intricately linked to angiogenesis by cooperating in VEGF/VEGFR signaling^1,47^. Moreover, c-MET has been found to be upregulated and in an activated state upon hypoxic conditions mediated by VEGFR inhibitors and pericyte loss in breast cancer models, including the 4T1 fat pad model^48^. Several studies have also reported that a combined VEGFR/c-MET inhibition reduced the angiogenic response in primary tumors and effectively decreased tumor progression^49–51^. Since many of the c-MET inhibitors initially in clinical development were reported to be nonselective, binding other receptor tyrosine kinases including VEGFR, this can at least in part account for their therapeutic effectiveness^7^. In marked contrast, OMO-1 treatment did not impact tumor angiogenesis, highlighting the superior selectivity of OMO-1 and indicating that an alternative mechanism probably underlies the hypoxia reduction in OMO-1-treated primary tumors.

Healthy blood vessels are lined by pericytes, which are responsible for vascular integrity and maturation. The leakiness of tumor blood vessels leads to increased interstitial pressure, hypoxia, impairs the delivery of therapeutics to the tumor, and stimulates metastasis as tumor cells are able to escape through these gaps in the vascular wall^52^. Normalization of the tumor vasculature by increasing pericyte coverage of the vascular wall can ameliorate these detrimental conditions^52–54^, and we hypothesize that OMO-1 could play a role in this process. In line with our hypothesis, the vascular bed was identified as a potential target for c-MET inhibition based on its detectable expression of c-MET in primary tumor sections. The increase in α-SMA^+^ pericytes covering CD31^+^ vessels upon c-MET inhibition by OMO-1 further verified OMO-1 treatment as indeed being dose dependently associated with vascular normalization. Consequently, cisplatin delivery was enhanced upon OMO-1 treatment, in turn leading to enhanced tumor cell death. Moreover, protein array analysis showed significant upregulation of four vascular integrity-associated proteins in primary tumor lysates of OMO-1-treated mice. More specifically, endoglin as a constituent of the TGF-β receptor complex in endothelial cells regulates interaction with pericytes^55,56^, FGF-1 as a heparin binding growth factor produced by endothelial cells, pericytes, and fibroblasts may mediate vascular maturation through regulation of vascular cadherin and integrin expression^57^, ICAM-1 as a vascular component for leukocyte infiltration regulates blood vessel permeability^58^, and MMP-3 as a pericyte-derived enzyme mediates extracellular matrix degradation for pericyte invasion^59^. Our data further suggest an imbalance between Ang-1 and -2 levels in favor of Ang-1, pericyte survival, and vessel normalization upon OMO-1 treatment in primary tumors. It should be emphasized that increased pericyte coverage and Ang-1 levels are unprecedented results for a c-MET inhibitor. Kobayashi et al. reported that HGF stimulates migration of smooth muscle cells/pericytes toward endothelial cells in response to Ang-1^60^. Liu et al. reported that c-MET is expressed in smooth muscle cells and pericytes and may be involved in pericyte migration to stabilize new blood vessels in atherosclerotic lesions^61^. Consequently, c-MET inhibition should rather attenuate pericyte recruitment and the likely vessel normalization with OMO-1 treatment is presumably due to other and/or compensatory mechanisms. EPT of tumor cells may provide an alternative explanation for the observed normalization effect. Tumor cells that are prone to spontaneous epithelial-to-mesenchymal transition can phenotypically and functionally resemble pericytes and thereby contribute to vascular stability and sustained tumor growth^33,34^. In line with this EPT concept, mesenchymal Hs578 TNBC cells have been reported to establish pericyte properties through expression of pericyte markers including α-SMA and PDGFR-β^62^. Yet, 4T1 cells treated with OMO-1 with or without cisplatin did not express pericyte markers, suggesting that EPT is unlikely to be responsible for the increase in α-SMA and PDGFR-β positivity seen in our study. Therefore, the underlying mechanism(s) for the suggested vessel maturation remain to be elucidated.

**Fig. 6:**
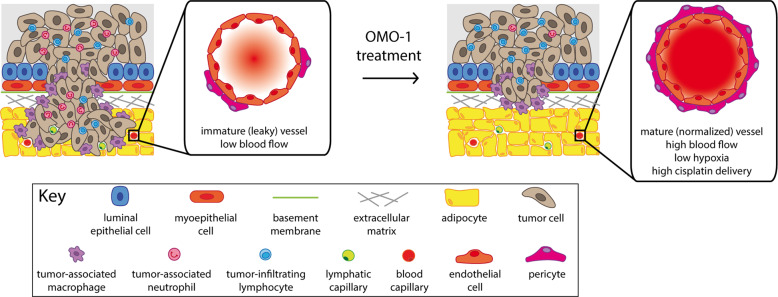
Working model for OMO-1 treatment in a 4T1-based intraductal model. Key findings upon treatment with OMO-1 are highlighted. More specifically, tumor growth is reduced, neutrophil influx is impaired (decreased MIP-2 levels and Ly6G positivity), and antitumor responses are more pronounced (increased levels of immunostimulatory and tumor suppressive proteins, decreased CD163 positivity). Leaky vessels are normalized to enhance the blood flow to the primary tumor (increased α-SMA, PDGFR-β, and Ang-1 positivity as well as enhanced α-SMA-covered CD31^+^ vessels), which in turn reduces tumor hypoxia and increases the delivery of chemotherapeutics (increased cisplatin DNA-adducts and cleaved caspase 3 levels).

In conclusion, our proof-of-concept study establishes OMO-1 as a potent and selective c-MET inhibitor with highly beneficial therapeutic effects as monotherapy and in combination with chemotherapy (cisplatin) in a 4T1-based immunocompetent intraductal mouse model for TNBC (Fig. 6). More specifically, we unequivocally demonstrate that OMO-1 treatment: (1) reduces malignant progression and provides additional antitumor effects to cisplatin in a model that is not c-MET oncogene addicted; (2) induces immunostimulatory and tumor suppressing expression profiles, further characterized by a favorable TME with significantly reduced numbers of TAMs in combination with cisplatin; and (3) reduces hypoxia, increases cisplatin delivery, and subsequent cell death in primary tumors potentially through vessel normalization. Future studies are warranted to validate our results in additional (TNBC) cell lines and to further delineate the mechanisms that underly the observed therapeutic efficacy of OMO-1.

## Methods

### 4T1 cell culture

4T1-luc mammary tumor cells that constitutively express the firefly luciferase gene and resemble the metastases observed in human TNBC (estrogen receptor-negative, progesterone receptor-negative, and human epidermal growth factor receptor 2-negative)^63,64^ were a kind gift from Prof. Clare Isacke (Breakthrough Breast Cancer Research Centre, London, UK). The cells were cultured at passage 8 in Dulbecco’s Modified Eagle’s Medium supplemented with 10% heat-inactivated fetal bovine serum, 100 U/ml penicillin, and 100 μg/ml streptomycin (Thermo Fisher Scientific, Waltham, MA, USA) in culture flasks at 37 °C and 5% CO_2_. Cell cultures were routinely checked for mycoplasma contamination using a mycoplasma detection kit (PlasmoTest^TM^; Invivogen, San Diego, USA). After washing with phosphate buffered saline (PBS) to remove dead particles, the cells were harvested using 0.25% trypsin-ethylenediaminetetraacetic acid (EDTA) (Sigma-Aldrich, Overijse, Belgium), washed through centrifugation (805 × *g* for 5 min), and resuspended in PBS. A Bürker chamber was used for counting of the cell numbers.

### Neutral red uptake assay on treated 4T1 cell cultures

4T1-luc cells at passage 12–13 were cultured at 3 × 10^4^ cells/ml in slide chambers (for immunocytochemistry) or in 24-well plates (for neutral red uptake assay and western blot analysis). After 24 h of incubation in 24-well plates and 48 h in slide chambers, when cell cultures reached 70% confluency, cells were treated with either DMSO (i.e., OMO-1 vehicle solution), 300 nM, 1 μM, or 3 μM OMO-1 in combination with or without cisplatin (Merck Millipore, Darmstadt, Germany). Cisplatin was dissolved in 0.9% sodium chloride solution (Sigma-Aldrich) and administered to the cell cultures at 2 μg/ml (for immunocytochemistry, neutral red uptake assay, and western blot analysis).

Neutral red uptake assays for estimation of cell viability/cytotoxicity were performed on cell cultures in 24-well plates following 48 h treatment. Untreated control wells were included, containing no cells (only culture medium) to correct possible absorption of neutral red to the plastic. Neutral red medium (0.33%, Sigma-Aldrich) was diluted 1:10 in colorless culture medium (without phenol red) and filtered using a 0.22 μm filter to remove precipitated dye crystals. Culture medium was aspirated from the 24-well plates and rinsed with culture medium without phenol red. One milliliter of the diluted neutral red medium (final concentration: 0.033%) was added to each well containing treated cells. After incubation of the plate for 2 h at 37°C and 5% CO_2_, neutral red medium was aspirated and the cells were rinsed with 300 μl PBS/well. Neutral red was extracted from the cells by adding 1 ml neutral red destaining solution per well and shaking the well plate rapidly on a plate shaker for at least 10 min. Finally, 150 μl of each 24-well plate was transferred to a 96-well plate. After removing air bubbles, the absorbances of neutral red extracts were measured at 540 nm on a microplate reader.

### Intraductal inoculations with 4T1 cells

Animal experiments were conducted according to Good Scientific Practice principles and approved by the Ethical Committee (EC) of the Faculty of Veterinary Medicine, Ghent University (EC 2018-37 and amendment EC 2018-65). In order to obtain full lactation, 8-week-old female BALB/c mice were mated with 8-week-old male BALB/c mice (both obtained from Envigo, Horst, The Netherlands) and pups were weaned 12–14 days post parturition. Lactating females were intraductally inoculated through the mammary teat canal of the third mammary gland pair 1 h after weaning using a 32-gauge blunt needle with 5 × 10^4^ 4T1-luc cells suspended in a 100 μl mixture of 1:10 PBS and Matrigel^®^ (Corning, Bedford, MA, USA). All materials used were precooled to avoid coagulation of the Matrigel^®^. A mixture of oxygen and isoflurane (2–3%) was used for inhalational anesthesia of the mice and a bolus of PBS-diluted Vetergesic (i.e., buprenorphine 10 µg/kg, Val d’Hony-Verdifarm NV, Belgium) was administered i.p. as analgesic prior to any surgical intervention.

### Treatment of tumor-bearing mice

As a positive control treatment, cisplatin was dissolved under sterile conditions in 0.9% sodium chloride solution in a dark recipient and 100 µl was administered i.p. every 5 days at 6 mg/kg. OMO-1 vehicle solution was freshly prepared every 3 days. In order to prepare the OMO-1 vehicle solution, methyl cellulose with viscosity 400 cPs (Alfa Aesar, Massachusetts, USA) was slowly brought in boiling dH_2_O at 0.5% (w/v) while stirring on a magnetic stirrer. The solution was subsequently sterilized in an autoclave and cooled to room temperature (RT) overnight (ON) while stirring on a magnetic stirrer. Before usage, the pH of the 0.5% methyl cellulose solution was adjusted to 2.5–3.5 using 1N HCl. The OMO-1 treatment solution was freshly prepared every day. In order to prepare it, the required volume of OMO-1 was weighed, 0.1 % (v/v) Tween-80 (Sigma-Aldrich) was added, and the contents were triturated to form a uniform mix using an appropriate pestle (pestle and microtube combo, VWR, Oud-Heverlee, Belgium). Next, 25% of the required volume of 0.5% methyl cellulose at pH 2.5–3.5 (i.e., the OMO-1 vehicle solution) was added and the mixture was triturated. Another 25% required volume of 0.5% methyl cellulose was then added for trituration and finally the remaining 50% of 0.5% methyl cellulose. The OMO-1 treatment solution was adjusted to pH 2.5–3.5, vortexed, and stored at 4 °C before usage. A stainless steel oral gavage needle was used to administer 100 µl of the OMO-1 treatment solution orally 2×/day (b.i.d.) from 21 days p.i. until 39 days p.i. at 3 mg/kg (low), 12.5 mg/kg (medium), or 25 mg/kg (high) concentration and the solution was vortexed between every dosing.

### Analysis of primary tumor growth and metastasis progression

The health of the animal was monitored weekly through measurement of the body weight (using a digital weighing scale) and body temperature (using a rectal temperature probe). Primary tumor volume was determined weekly as a measurement of primary tumor growth using a digital caliper. In vivo imaging of the 4T1-luc-derived bioluminescence in primary tumors was also determined at 7, 21, and 39 days p.i. using the IVIS lumina II system (PerkinElmer, Zaventem, Belgium). Mice were therefore injected with 200 µl D-luciferin suspended in PBS (2 mg/100 µl; Gold Biotechnology, St. Louis, MO) and images were acquired 10 min later under inhalation anesthesia (mixture of oxygen and isoflurane (2–3%)). At 39 days p.i., mice were injected i.p. with 60 mg/kg pimonidazole hydrochloride (Hypoxyprobe, Burlington, Massachusetts, USA) in 0.9% NaCl, which allows for hypoxia assessment on isolated primary tumors through immunohistochemistry. Primary tumors, spleens, and metastases-bearing organs, including axillary lymph nodes, lungs, and liver, were isolated 1–2 h later by first sedating the mice using a mixture of 100 mg/kg ketamine (Ketamidor, Ecuphar nv/sa, Oostkamp, Belgium) and 10 mg/kg xylazine (xylazini hydrochloridum, Val d’Hony-Verdifarm, Beringen, Belgium) followed by sacrification of the mice through cervical dislocation. Primary tumors and spleens were weighed using a digital weighing scale following isolation. IVIS measurements and subsequent analysis using the living image analysis software 3.2 allowed to quantify 4T1-luc-derived bioluminescence in the primary tumors and isolated organs.

### Measurement of cytokine and protein levels

Primary tumors and spleens were homogenized, mixed with 300 μl lysis buffer supplemented with protease inhibitors (1% Nonidet P-40, 10 mM Tris-HCl at pH 7.4, 200 mM NaCl, 5 mM EDTA, 10% glycerol, 100 μM phenylmethylsulfonyl, 1 mM oxidized L-glutathione (all from Sigma-Aldrich), 0.15 μM aprotinin, and 2.1 μM leupeptin (Roche, Mannheim, Germany)), and lysed ON at −20 °C. Samples were centrifuged the following day at 17,000 × *g* for 1 h at 4 °C and the lysates were obtained from the supernatant. Protein concentrations in the latter were measured using the Bradford Protein Assay (Bio-Rad, Hercules, CA) and spectrophotometry (Multiskan GO, Thermo Scientific) and diluted to 5 μg/μl with lysis buffer. Blood was harvested through cardiac puncture, clotted, and centrifuged at 17,000 × *g* for 1 h at 4 °C to obtain serum. A Proteome Profiler Mouse XL Cytokine Array (Bio-Techne, Minneapolis, MN, USA) using 200 µg primary tumor lysates was used according to the manufacturer’s instructions. A ChemiDoc MP Imaging System (Bio-Rad) was used for detection and the data were analyzed in ImageJ. Relative protein concentrations were obtained by subtraction of the background staining and subsequent normalization to positive controls on the membrane. Ten cytokines (BAFF, G-CSF, IFN-γ, IL-1β, IL-4, IL-6, IL-10, MCP-1, MIP-2, and TNF-α) and two additional immune checkpoint proteins (CTLA-4 and PD-1) were quantified in primary tumor lysates (50 μg protein) using the Luminex Multiplex Assay (ProcartaPlex from Thermo Fisher Scientific) according to the manufacturer’s instructions. ELISA was used to measure the levels of CHI3L1 and LCN2 (Mouse Quantikine ELISA Kit, Bio-Techne) in primary tumor lysates, sera and spleen lysates, Tie-2 levels (Mouse Quantikine ELISA Kit, Bio-Techne) in both primary tumor lysates and sera, and granzyme B (Mouse precoated ELISA Kit, Thermo Fisher Scientific), TGF-β1 levels (Mouse uncoated ELISA Kit, Thermo Fisher Scientific), as well as VEGF levels (Mouse Quantikine ELISA Kit, Bio-Techne) in primary tumor lysates according to the manufacturer’s instructions. For ELISA measurements, absorbances at 450 and 550 nm (the latter subtracted from the former for correction purposes) were obtained using a microplate reader (Multiskan GO, Thermo Scientific) and analyses were performed with Deltasoft.

### Histology and immunohisto/cytochemistry

Isolated tissues (primary tumors, axillary lymph nodes, lungs, and livers) were fixed in buffered 3.5% formaldehyde for 24 h at RT and embedded in paraffin wax. Sections of 5 µm were deparaffinized, hydrated, and stained with H&E. Sections were then dehydrated and mounted.

For immunohistochemical stainings, antigen retrieval was performed on deparaffinized sections with Tris-EDTA buffer (10 mM Tris, 1 mM EDTA (Thermo Fisher Scientific); for cytokeratin 5, CTLA-4, c-MET, and pan-cytokeratin), pH 9 or citrate buffer (10 mM tri-sodium citrate (Santa Cruz Biotechnology, Heidelberg, Germany); for all other targets), pH 6 with 0.05% Tween-20 (Sigma-Aldrich) at 95 °C for 30 min using a pressurized Decloaking Chamber NxGen (Biocare Medical, CA, USA). The slides were then cooled down to RT for 30 min, treated with 0.06% (for CD8a) or 3% (all other targets) H_2_O_2_ in methanol for 10 min to block endogenous peroxidase activity and with levamisole (2 mM in dH_2_O, only for dual staining immunohistochemistry) to block alkaline phosphatase activity, followed by a serum-free protein block (Dako, Heverlee, Belgium) treatment for 10 min to block nonspecific binding sites. For immunocytochemical staining, culture medium was aspirated from the slide chambers, cultured cells were fixed using 3.7% paraformaldehyde and permeabilized by applying ice cold methanol for 4 min. The slides were then rinsed with Tris-buffered saline (TBS, Biocare Medical) and incubated with 5% BSA (Sigma-Aldrich) in TBS for 30 min.

The next steps were identical for immunohistochemical and immunocytochemical staining. More specifically, the slides were stained with primary antibodies diluted in Antibody Diluent (Dako) for 1 h at RT and secondary antibodies (ready-to-use) for 30 min at RT. The used primary antibodies and dilutions were: anti-cytokeratin 5 (1:100, clone EP1601Y, Abcam, Cambridge, UK), anti-Ki67 (1:50, clone SP6; Thermo Fisher Scientific), anti-pan-cytokeratin (1:100, polyclonal, Abcam), anti-CD11b (1:4000, clone EPR1344, Abcam), anti-CD163 (1:500, clone EPR19518, Abcam), anti-Ly6G (1:1000; clone 1A8; BioLegend, CA, USA), anti-CD8a (1:50, clone 4SM15, Thermo Fisher Scientific), anti-granzyme B (1:1000, polyclonal, Abcam), anti-PD-1 (1:1000, clone EPR20665, Abcam), anti-CTLA-4 (1:500, clone CAL49, Abcam), FITC-conjugated anti-pimonidazole (1:25; clone 4.3.11.3; Hypoxyprobe), anti-CAIX (1:1000; clone NB100–417; Novus Biologicals, Littleton, CO, USA), anti-CD31 (1:2000; clone EPR17259; Abcam), anti-α-SMA (1:2000, clone EPR5368, Abcam), anti-PDGFR-β (1:500, clone Y92, Abcam), anti-Ang-1 (1:500, polyclonal, Abcam), anti-cisplatin DNA-adducts (1:50, clone ICR4, Sigma-Aldrich), and anti-cleaved caspase 3 (1:1000, clone 5A1E, Cell Signaling Technology, Leiden, The Netherlands). The used secondary antibodies were: Rat-on Mouse HRP-Polymer (Biocare Medical) for Ly6G, CD8a, and cisplatin DNA-adducts, and Dako EnVision+ Rabbit (Dako) for cytokeratin 5, Ki67, pan-cytokeratin, CD11b, CD163, granzyme B, PD-1, CTLA-4, CAIX, CD31, α-SMA, PDGFR-β, Ang-1, and cleaved caspase 3. For visualization of HRP-positive staining, slides were treated with a 3,3′-diaminobenzidine (DAB)-containing buffer (Dako) for 10 min at RT. Counterstaining with hematoxylin was applied for 5 min at RT, followed by dehydration of the tissue slides. Pimonidazole stainings did not require a secondary antibody and was counterstained with DAPI (0.4 µg/ml; Sigma-Aldrich) for 10 min at RT. Mounting of the DAB-treated and pimonidazole-stained slides was performed with Tissue-Tek Glas mounting medium and antifading DABCO mounting medium, respectively. For double staining immunohistochemistry, rat anti-CD31 (1:20, clone SZ31, Dianova, Hamburg, Germany) and rabbit anti-α-SMA (1:2000, clone EPR5368, Abcam) were simultaneously applied for 1 h at RT and the DoubleStain IHC Kit: R&Rt on Human/Mouse Tissue (Green/HRP and AP/Red) was used for the subsequent steps according to the manufacturer’s instructions. Hematoxylin counterstain was not performed to allow easier detection of codistribution (dark blue). All rinsing steps were performed with TBS and were applied three times for 2 min at RT between every incubation. Slides were incubated on an orbital shaker at 20 rpm in a closed microscope box with TBS-wetted tissue paper for all blocking, rinsing, and staining steps. Quantification of positive staining was established using color deconvolution (for DAB and hematoxylin counterstaining) or 8-bit image conversion (for pimonidazole stainings) followed by automatic counting in ImageJ. Ki67 proliferation index was calculated using ImageJS by capturing all nuclei and Ki67^+^ nuclei from Ki67 stained primary tumor sections^65^.

### Western blot analyses

Primary tumor and 4T1 cell culture lysates were loaded on 12% Mini-PROTEAN TGX Precast Protein Gels (Bio-Rad, CA, USA) at 25 µg per lane. Lysates from treated 4T1 cultures were prepared with the same lysis buffer used to lyse isolated tissues, but was additionally supplemented with phosphatase inhibitor cocktail 1 (Sigma-Aldrich) to ensure visualization of phosphorylated proteins. Lysates from treated NCI-H441 cultures were prepared with a heated buffer (10 mM Tris pH 7.4, 1 mM sodium orthovanadate, and 1% SDS) and diluted in commercial lysis buffer (MesoScale Met Duplex Kit) and loaded on a Nu-Page gel (Invitrogen, CA, USA) at 40 µg per lane. Proteins in all gels were electrophoretically separated and transferred on to 0.45 µm nitrocellulose membrane (Bio-Rad). All further steps were performed on a shaking platform. Either freshly prepared blocking buffer (TBS with 0.1% Tween-20 and 5% nonfat dry milk) or commercial odyssey blocking buffer (only for NCI-H441 lysates; Li-COR bioscience, Bad Homburg, Germany) was applied for 1 h at RT and membranes were then incubated with primary antibody (diluted in blocking buffer) ON at 4 °C. The used primary antibodies and dilutions were: anti-c-MET (1:1000, clone 25H2, Cell Signaling Technology; or 1:1000, clone EP1454Y, Abcam), anti-phospho-c-MET (Tyr1230/1234/1235; 1:1000, polyclonal, Thermo Fisher Scientific), anti-phospho-Gab2 (Tyr452; 1:1000, polyclonal, Cell Signaling Technology), anti-phospho-Akt (Ser473; 1:2000, clone D9E or 1:1000, polyclonal for NCI-H441 lysates, both from Cell Signaling Technology), anti-phospho-MEK1/2 (Ser217/221, 1:1000, polyclonal, Cell Signaling Technology), anti-phospho-p44/42 MAPK (Erk1/2; Thr202/Tyr204; 1:2000, clone D13.14.4E or 1:1000, clone E10 for NCI-H441 lysates, both from Cell Signaling Technology), anti-β-actin (1:5000, clone AC-15, Sigma-Aldrich), anti-Ang-2 (1:500, polyclonal, Novus Biologicals), anti-cleaved caspase 3 (1:1000, clone 5A1E, Cell Signaling Technology), and anti-glyceraldehyde 3-phosphate dehydrogenase (GAPDH; 1:5000, clone EPR16891, HRP-conjugated, Abcam). Washing of the membranes was performed the following day with blocking buffer (three times, 5 min each) prior to incubation of the membranes with secondary antibody (diluted in blocking buffer) for 1 h at RT. The used secondary antibodies were as follows: anti-mouse IgG, HRP-linked antibody (1:3000, Cell Signaling Technology) for c-MET (Cell Signaling Technology); donkey anti-rabbit IgG HRP-conjugated polyclonal antibody (1:10000, Thermo Fisher Scientific) for c-MET (Abcam), phospho-Akt (clone D9E), phospho-p44/42 MAPK (Erk1/2, clone D13.14.4E), Ang-2 and cleaved caspase 3; goat anti-mouse IgG (H + L) Alexa Fluor 680-conjugated polyclonal antibody (1:1000, Thermo Fisher Scientific) for c-MET (Cell Signaling Technology, only for NCI-H441 lysates) and phospho-p44/42 MAPK (Erk1/2, clone E10); goat anti-rabbit IgG (H + L) Alexa Fluor 680-conjugated polyclonal antibody (1:1000, Thermo Fisher Scientific) for phospho-c-MET, phospho-Gab2 (polyclonal), phospho-Akt (polyclonal), phospho-MEK1/2 (polyclonal); and donkey anti-mouse IgG (H + L) IRDye 800-conjugated polyclonal antibody (1:1000, Rockland Inc., PA, USA) for β-actin. Following another washing step with blocking buffer (three times, 5 min each) and with dH_2_O (one time, 5 min), protein bands were detected on the membranes using the SuperSignal West Femto Kit (Thermo Fisher Scientific) on the ChemiDoc MP Imaging System or scanned on the odyssey (only for NCI-H441 lysates). Pageruler Prestained Protein Ladder (Thermo Fisher Scientific), Precision Plus Protein Dual Color Standards (Bio-Rad), and MagicMark (Invitrogen) provided molecular weight markers on the membranes and allowed to determine the size of the detected protein bands. Quantification of the signals was performed with ImageJ. Full images of all western blots and corresponding molecular weight markers are shown in the Supplementary information (Supplementary Figs. 12–17).

### Statistical analyses

Statistics were performed using Prism (GraphPad). Data normalization was performed through log_10_ normalization if necessary. *P* values were calculated by one-way analysis of variance tests followed by Newman–Keuls or Tukey’s post hoc test for multiple comparisons.

### Reporting summary

Further information on research design is available in the Nature Research Reporting Summary linked to this article.

## Supplementary information

Supplementary information

Reporting Summary Checklist

## Data Availability

The data generated and analyzed during this study are described in the following metadata record: 10.6084/m9.figshare.13615397^66^. The histology and immunohisto/cytochemistry, spreadsheets, and western blot analysis data are not publicly available for the following reason: a patent application on OMO-1 in non-c-MET addicted tumors is still pending. However, the data can be made available upon reasonable request to the corresponding author. A comprehensive list of the datafiles underlying each of the figures and supplementary figures is shared in the metadata record.
