# Supplementary material for: OMO-1 reduces progression and enhances cisplatin efficacy in a 4T1-based non-c-MET addicted intraductal mouse model for triple-negative breast cancer
Source: NPJ Breast Cancer. 2021 Mar 17;7:27. doi: 10.1038/s41523-021-00234-8 (PMC7969607; doi:10.1038/s41523-021-00234-8)
Supplement: Supplementary file 1 — Supplementary information [file 41523_2021_234_MOESM1_ESM.pdf]

Supplementary information

OMO-1 reduces progression and enhances cisplatin efficacy in a 4T1-based non c-MET addicted intraductal mouse model for triple-negative breast cancer

Authors and affiliations: Jonas Steenbrugge[1,2], Niels Vander Elst[1], Kristel Demeyere[1], Olivier De Wever[2,3], Niek N. Sanders[2,4], Wim Van Den Broeck[5], Eric Ciamporcero[6], Timothy Perera[6] and Evelyne Meyer[1,2]

[1]Laboratory of Biochemistry, Department of Pharmacology, Toxicology and Biochemistry, Faculty of Veterinary Medicine, Ghent University, Merelbeke, Belgium

[2]Cancer Research Institute Ghent (CRIG), Ghent, Belgium

[3]Laboratory of Experimental Cancer Research, Department of Human Structure and Repair, Ghent University, Ghent, Belgium.

[4]Laboratory of Gene Therapy, Department of Nutrition, Genetics and Ethology, Faculty of Veterinary Medicine, Ghent University, Merelbeke, Belgium

[5]Department of Morphology, Faculty of Veterinary Medicine, Ghent University, Merelbeke, Belgium

[6]OCTIMET Oncology NV, Beerse, Belgium

Supplementary Fig. 1

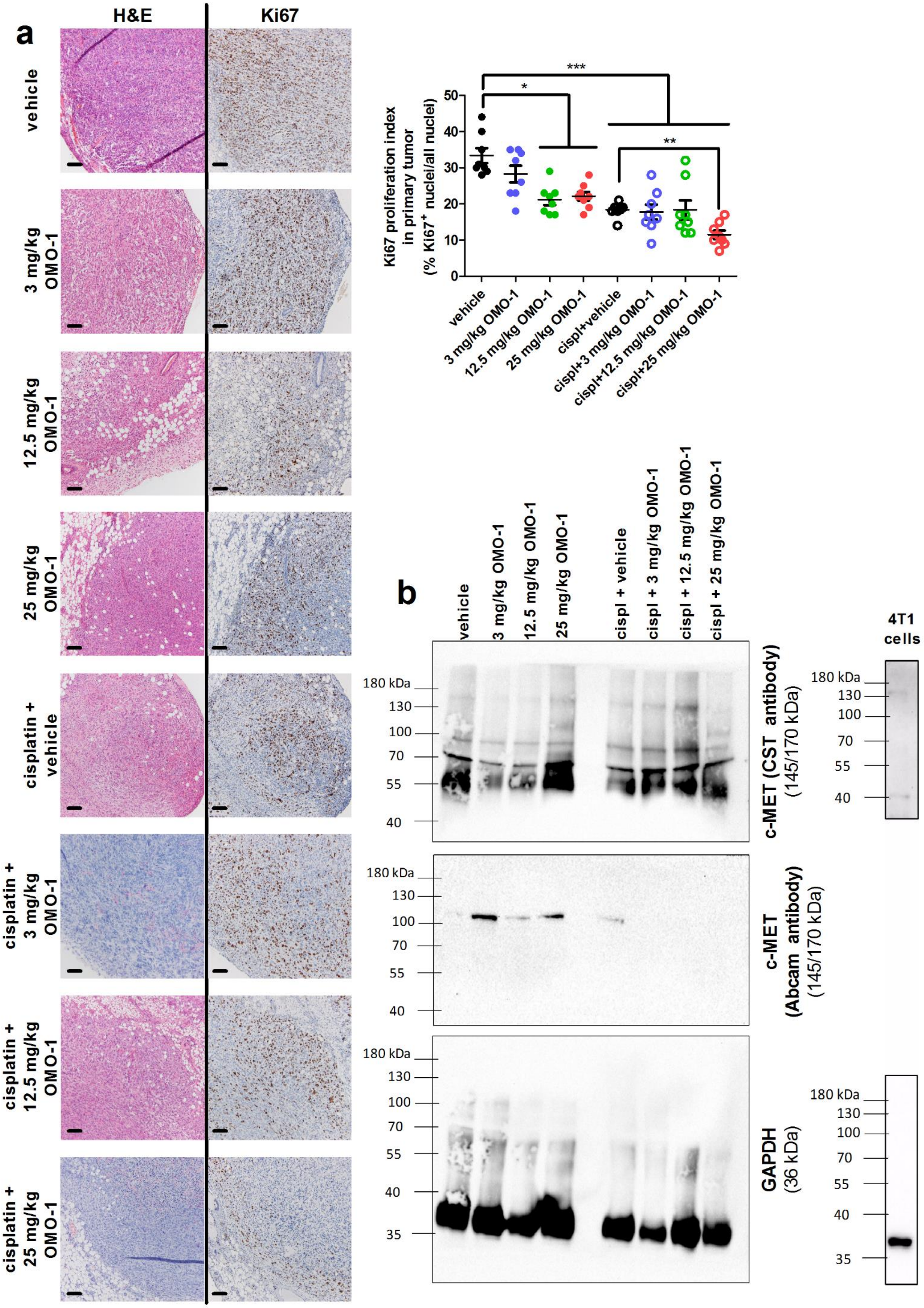

Supplementary Fig. 1. OMO-1 treatment decreases tumor proliferation upon minimal c-MET expression in a 4T1-based intraductal model.

(**a**) H&E histology and immunohistochemistry for the cell proliferation marker Ki67 on paraffin sections of primary tumors from the different treatment groups at 39 d p.i. (n = 8; 2 slides per treatment group for each marker with 4 images per slide). The Ki67 proliferation index determines the amount of $Ki67^+$ nuclei relative to all nuclei. Scale bars = 100 µm. (**b**) Western blot for c-MET and GAPDH (loading control) in lysates of primary tumors from the different treatment groups at 39 d p.i. and lysates of cultured 4T1 tumor cells. Data in panel **a** are presented as the means +/- SEM. *: $P < 0.05$, ***: $P < 0.001$.

Supplementary Fig. 2

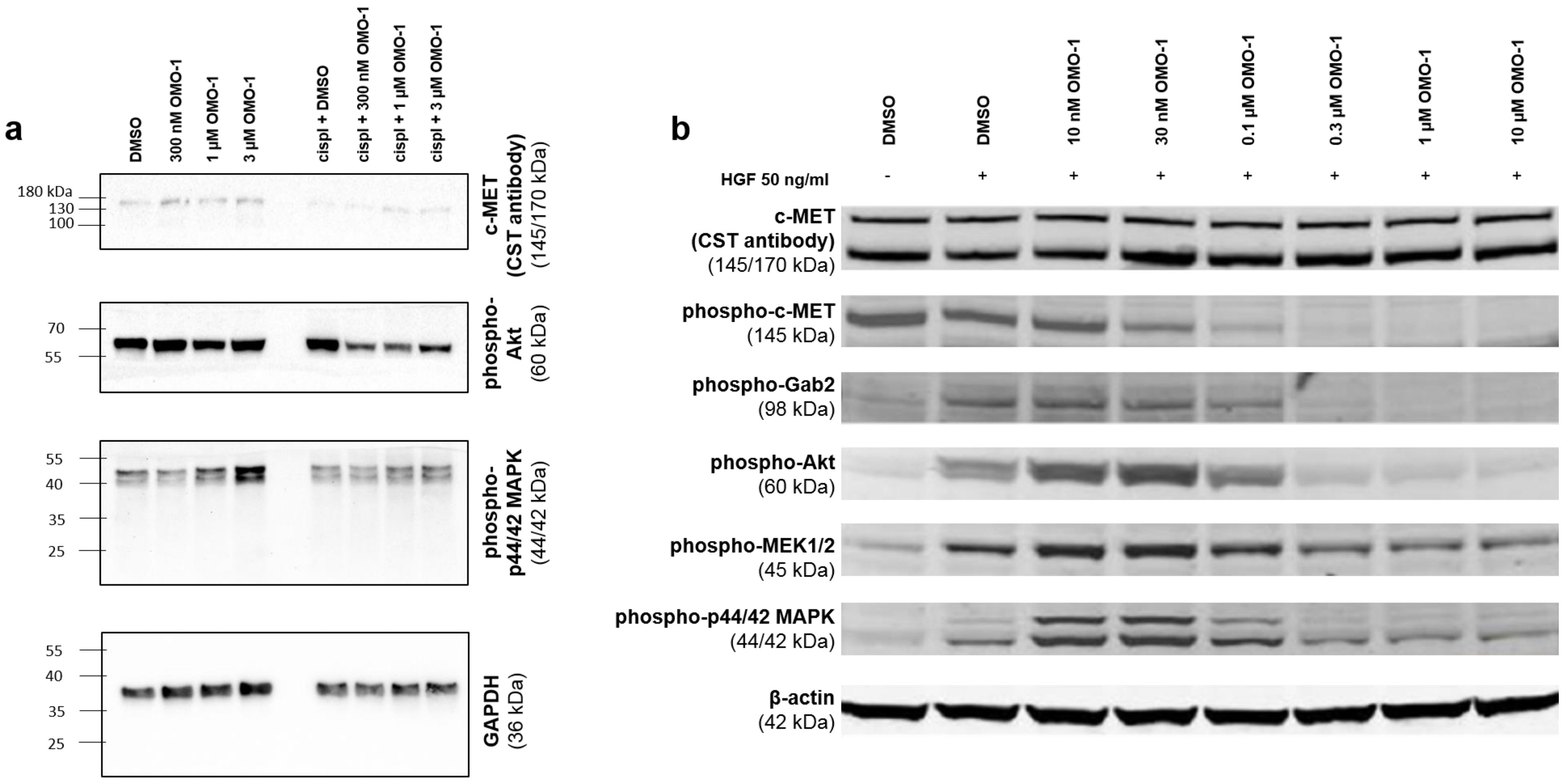

Supplementary Fig. 2. OMO-1 treatment does not affect phosphorylation of downstream c-MET signaling effectors in 4T1 cells *in vitro*.

(**a**) Western blot for c-MET, phospho-p44/42 MAPK (Erk1/2), phospho-Akt and GAPDH (loading control) in lysates of cultured 4T1 cells following 48 h treatment with DMSO, OMO-1, cisplatin + DMSO and cisplatin + OMO-1. (**b**) Western blot for c-MET, phospho-c-MET, phospho-Gab2, phospho-Akt, phospho-MEK1/2, phospho-p44/42 MAPK (Erk1/2) and β-actin (loading control) in lysates of c-MET amplified NCI-H441 non-small cell lung carcinoma cells following treatment with DMSO and increasing doses of OMO-1 for 20 min and with prior c-MET stimulation using HGF. All blots include samples from the same experiment.

Supplementary Fig. 3

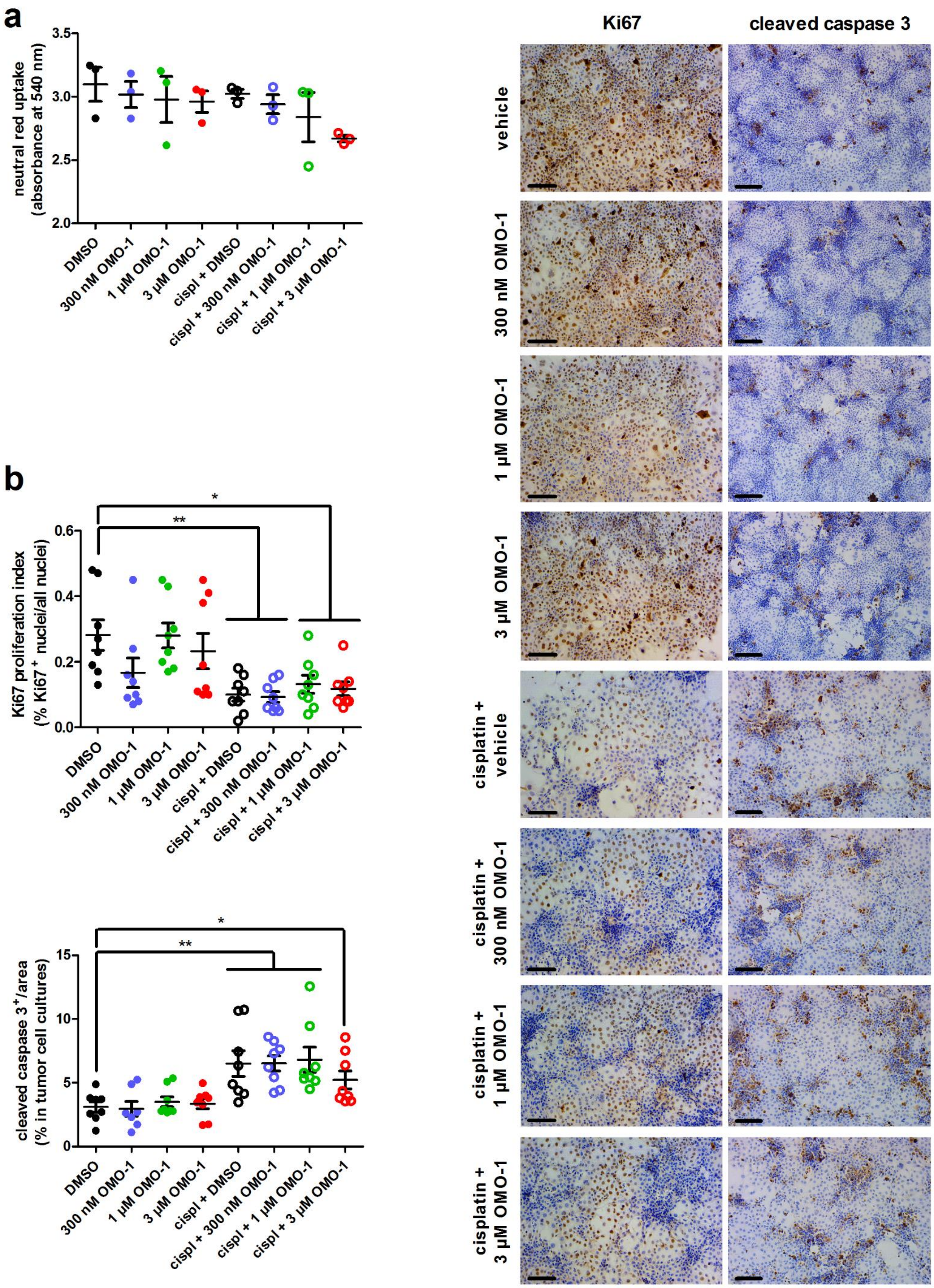

Supplementary Fig. 3. OMO-1 treatment does not affect *in vitro* 4T1 cellular growth, proliferation and cell death.

(**a**) Neutral red uptake results of cultured 4T1 tumor cells following 48 h treatment with DMSO, OMO-1, cisplatin + DMSO and cisplatin + OMO-1 (n = 3 for each treatment group). (**b**) Quantification of Ki67 proliferation index and cleaved caspase 3 immunocytochemical stainings of cultured 4T1 tumor cells following 48 h treatment (n = 8; 2 slides per treatment group for each marker with 4 images per slide). Scale bars = 200 µm. Data are presented as the means +/- SEM. *: $P < 0.05$, **: $P < 0.01$.

Supplementary Fig. 4

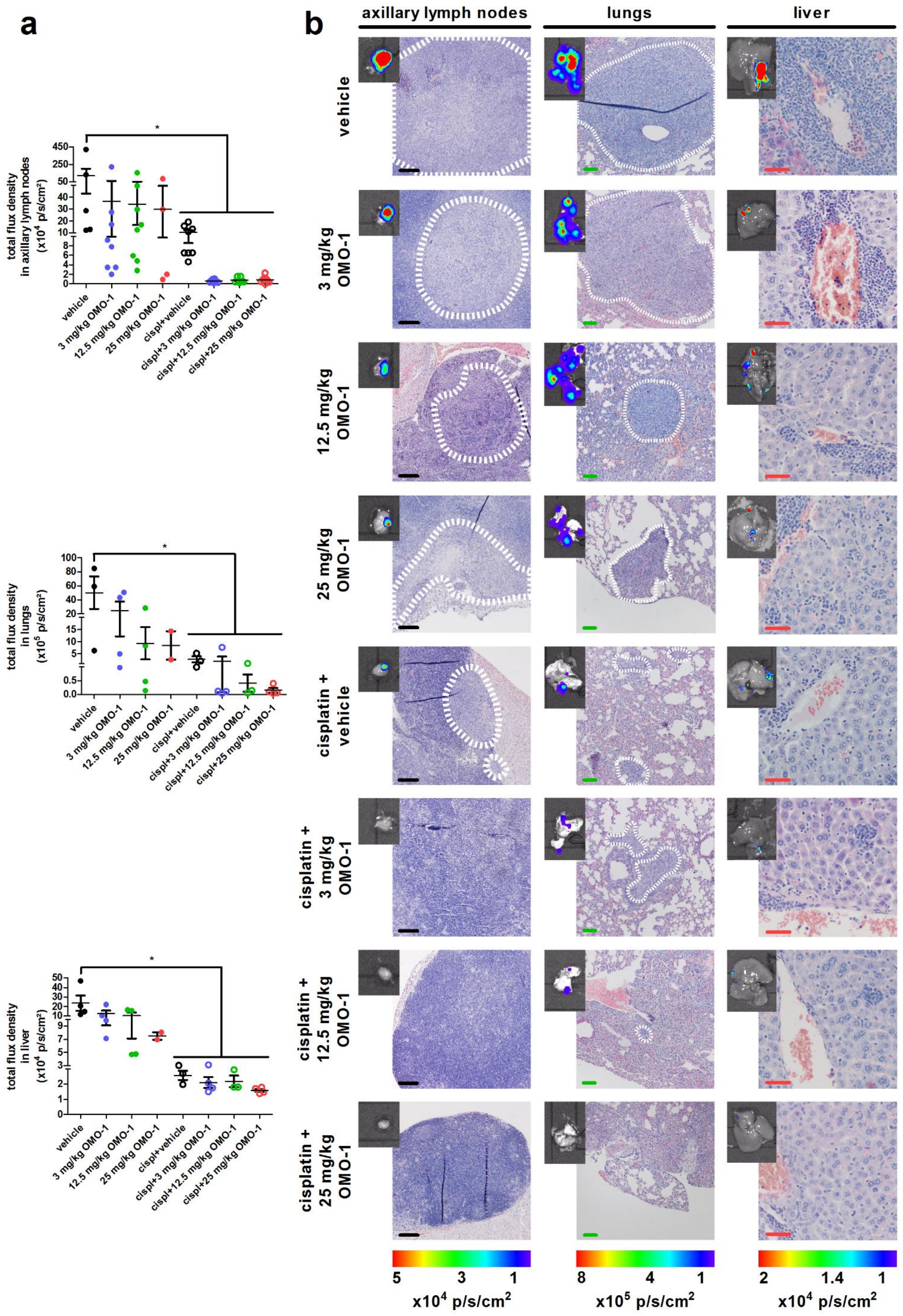

Supplementary Fig. 4. OMO-1 treatment decreases primary tumor metastasis-derived bioluminescent signals in a 4T1-based intraductal model.

(**a**) Quantification of 4T1-derived bioluminescent signals in the different treatment groups at 39 d p.i. based on *ex vivo* imaging in isolated axillary lymph nodes, lungs and liver (in p/s/cm²) (axillary lymph node numbers: n = 5 for vehicle, n = 4 for 25 mg/kg OMO-1, n = 6 for cisplatin + 12.5 mg/kg OMO-1, n = 8 for all other treatment groups; lung and liver numbers: n = 3 (lungs) and 4 (liver) for vehicle, n = 2 for 25 mg/kg OMO-1, n = 3 for cisplatin + vehicle and cisplatin +12.5 mg/kg OMO-1, n = 4 for all other treatment groups). (**b**) Representative images of the *ex vivo* bioluminescence signals in axillary lymph nodes, lungs and liver at 39 d p.i., and H&E histology of metastasized tumor cells/infiltrated cells (only for the liver) in these organs. White dashed lines indicate tumor tissue. Black scale bars = 200 µm, green scale bars = 100 µm, red scale bars = 50 µm. Data in panel **a** are presented as the means +/- SEM. *: $P < 0.05$.

Supplementary Fig. 5

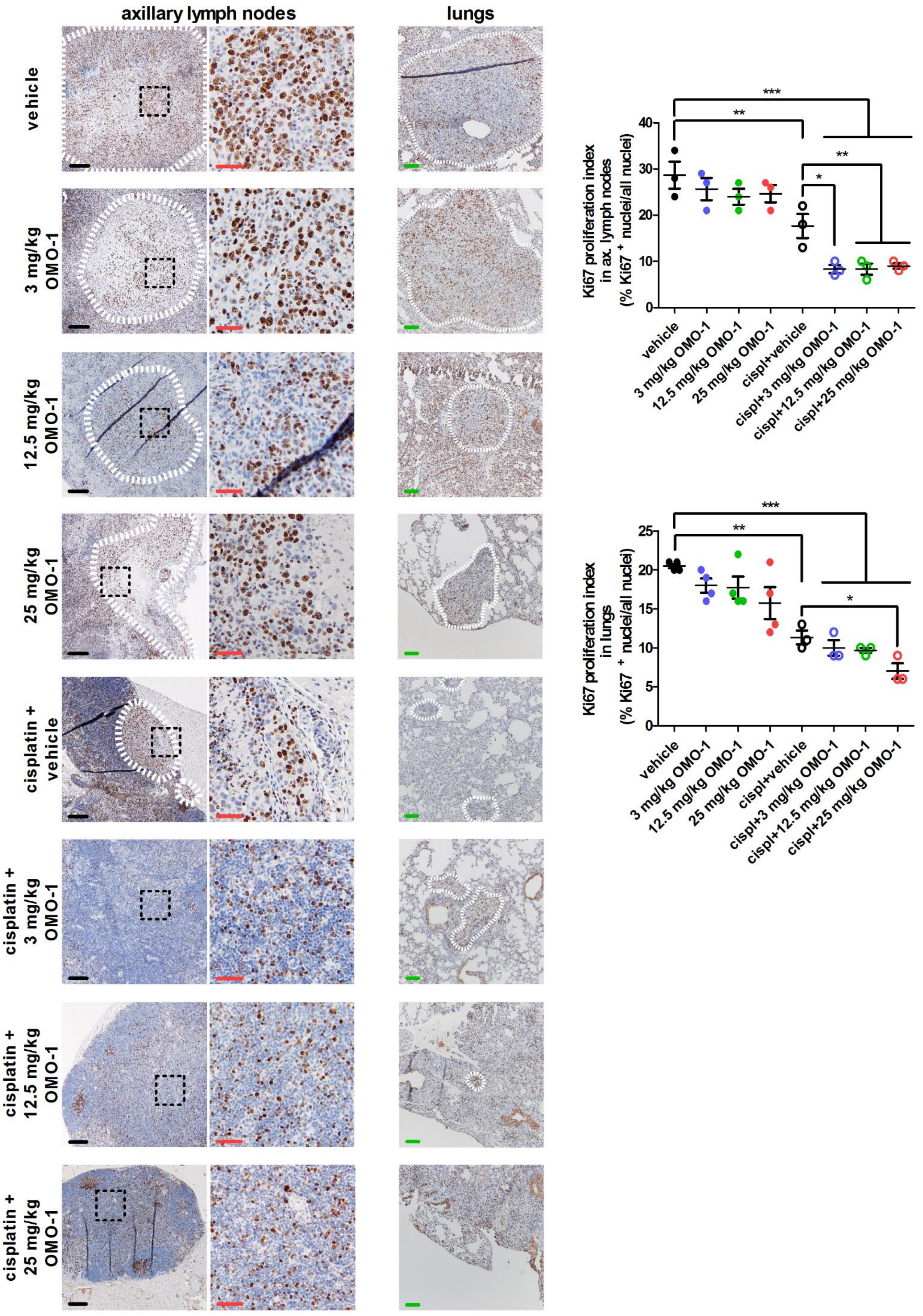

Supplementary Fig. 5. Ki67 staining-based confirmation of decreased axillary lymph node and lung metastasis upon OMO-1 treatment in a 4T1-based intraductal model.

Immunohistochemistry for the cell proliferation marker Ki67 performed on paraffin sections of axillary lymph nodes, and lungs from the different treatment groups at 39 d p.i. (n = 8; 2 slides per treatment group with 4 images per slide). Ki67 proliferation indices (number of $Ki67^+$ nuclei relative to all nuclei) were determined. White dashed lines indicate tumor tissue. Dashed inserts highlight a detailed image of Ki67 staining in axillary lymph nodes. Black scale bars = 200 µm, green scale bars = 100 µm, red scale bars = 50 µm. Data are presented as the means +/- SEM. **: $P < 0.01$, ***: $P < 0.001$.

Supplementary Fig. 6

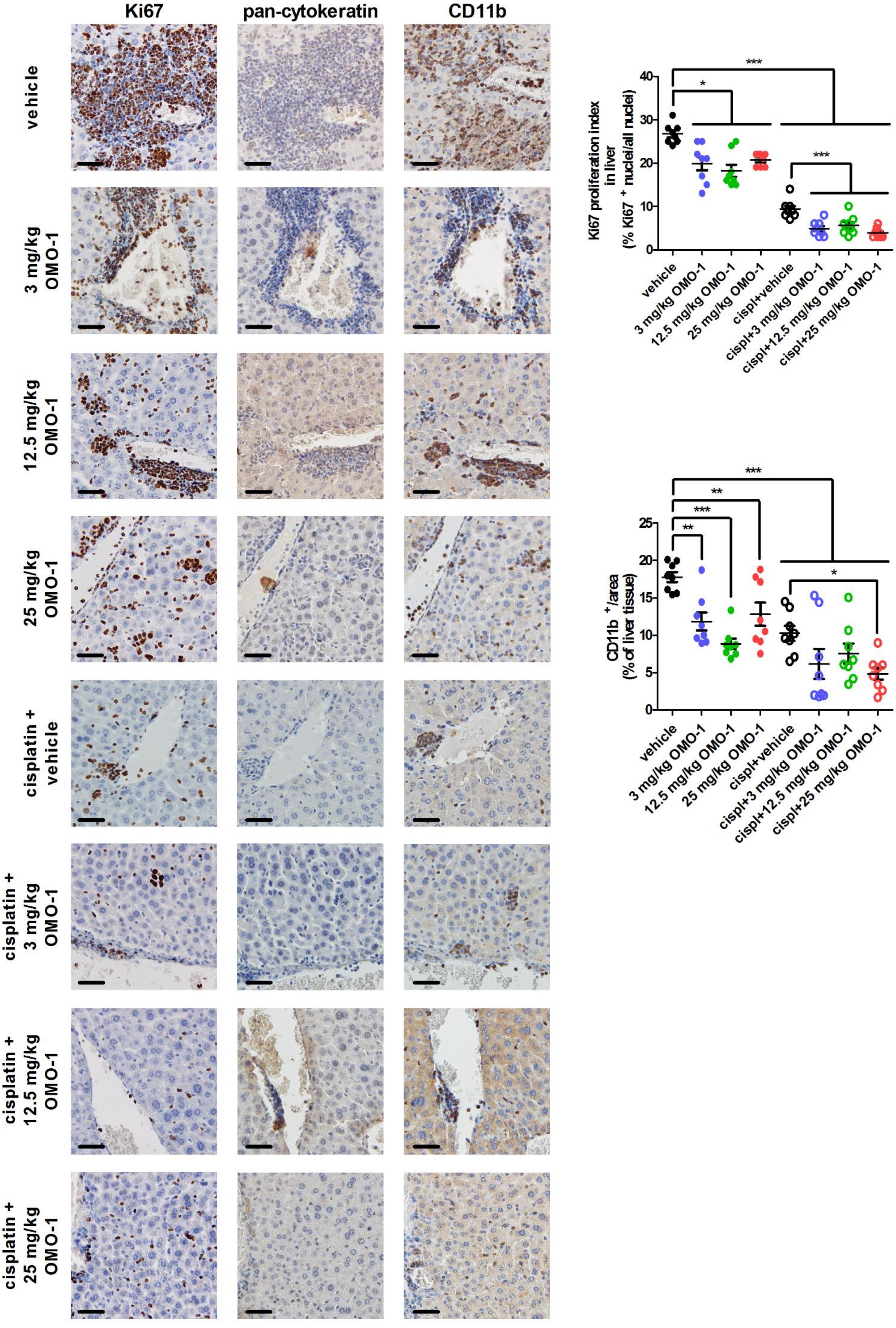

Supplementary Fig. 6. Liver tissue of the 4T1-based intraductal model is characterized by extramedullary hematopoiesis which is decreased by OMO-1 treatment.

Immunohistochemistry for the cell proliferation marker Ki67, a pan-cytokeratin marker, and the pan-myeloid cell marker CD11b performed on serial paraffin sections of liver tissue from the different treatment groups at 39 d p.i. Ki67 proliferation indices (number of $Ki67^+$ nuclei relative to all nuclei) and CD11b staining quantifications were determined (n = 8; 2 slides per treatment group with 4 images per slide).

Supplementary Fig. 7

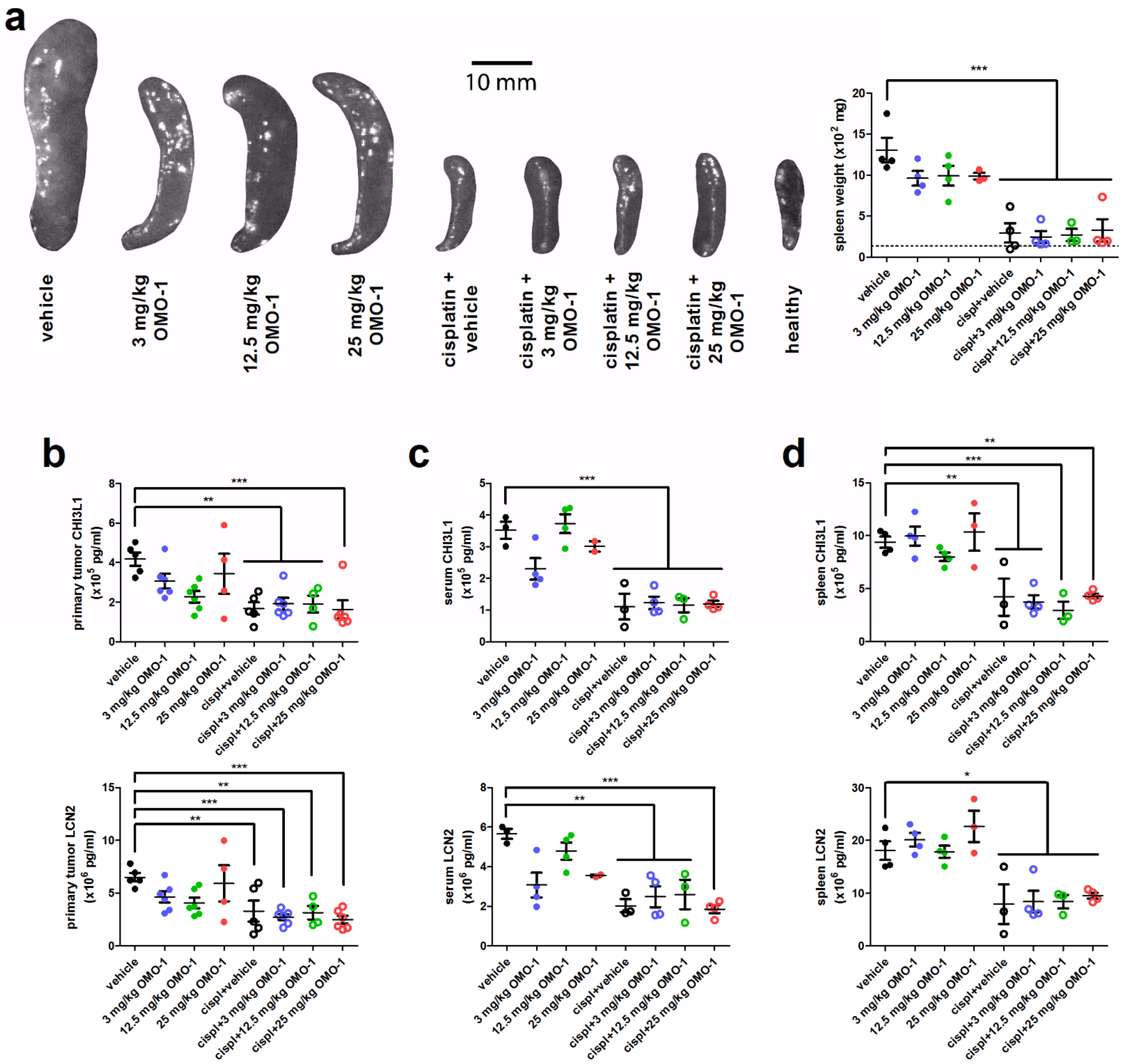

Supplementary Fig. 7. Cisplatin with or without OMO-1 reduces splenomegaly and immune-related biomarkers in a 4T1-based intraductal model.

(**a**) Representative images and weight measurements (right graph) of spleens isolated from the different treatment groups at 39 d p.i. (n = 3 for 25 mg/kg OMO-1 and cisplatin + 12.5 mg/kg OMO-1, n = 4 for all other treatment groups). The spleen from a healthy BALB/c mouse is shown for comparison. The dotted line in the right graph demonstrates the mean spleen weight of 6 healthy BALB/c mice. (**b**) Primary tumor CHI3L1 and LCN2 levels from the different treatment groups at 39 d p.i. (n = 5 for vehicle and cisplatin + vehicle treatment group, n = 4 for 25 mg/kg OMO-1 and cisplatin + 12.5 mg/kg OMO-1, n = 6 for all other treatment groups). (**c**) Serum CHI3L1 and LCN2 levels from the different treatment groups at 39 d p.i. (n = 3 for vehicle, cisplatin + vehicle and cisplatin + 12.5 mg/kg OMO-1, n = 2 for 25 mg/kg OMO-1, n = 4 for all other treatment groups). (**d**) Spleen CHI3L1 and LCN2 levels from the different treatment groups at 39 d p.i. (n = 3 for 25 mg/kg OMO-1, cisplatin + vehicle and cisplatin + 12.5 mg/kg OMO-1, n = 4 for all other treatment groups). Data are presented as the means +/- SEM. *: $P < 0.05$, **: $P < 0.01$, ***: $P < 0.001$.

Supplementary Fig. 8

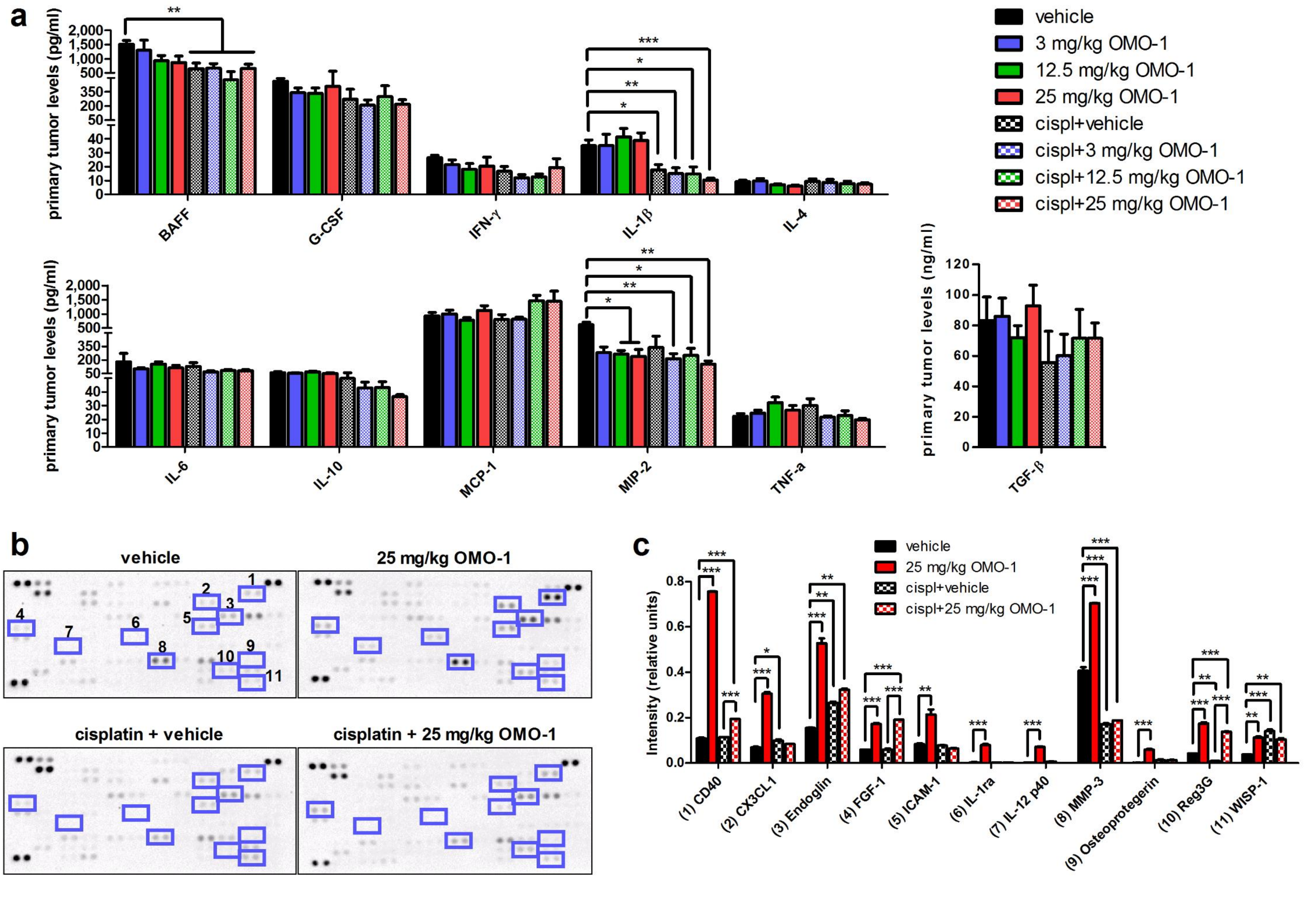

Supplementary Fig. 8. Tumor cytokine levels and protein array analysis upon OMO-1, cisplatin and combination treatment in a 4T1-based intraductal model.

(**a**) Primary tumor cytokine levels at 39 d p.i. (n = 5 for vehicle and cisplatin + vehicle, n = 4 for 25 mg/kg OMO-1 and cisplatin + 12.5 mg/kg OMO-1, n = 6 for all other treatment groups). (**b**) Protein array blot from lysates of 4T1 primary tumors at 39 d p.i. treated with vehicle, 25 mg/kg OMO-1, cisplatin + vehicle and cisplatin + 25 mg/kg OMO-1. Detectable proteins that showed a significantly different expression between the treatment groups are highlighted. (**c**) Quantification of protein array results revealing upregulation of the 11 selected proteins upon treatment with OMO-1, especially as monotherapy (each treatment group containing 2 replicates). Data in panel **a** and **c** are presented as the means +/- SEM *: $P < 0.05$, **: $P < 0.01$, ***: $P < 0.001$.

Supplementary Fig. 9

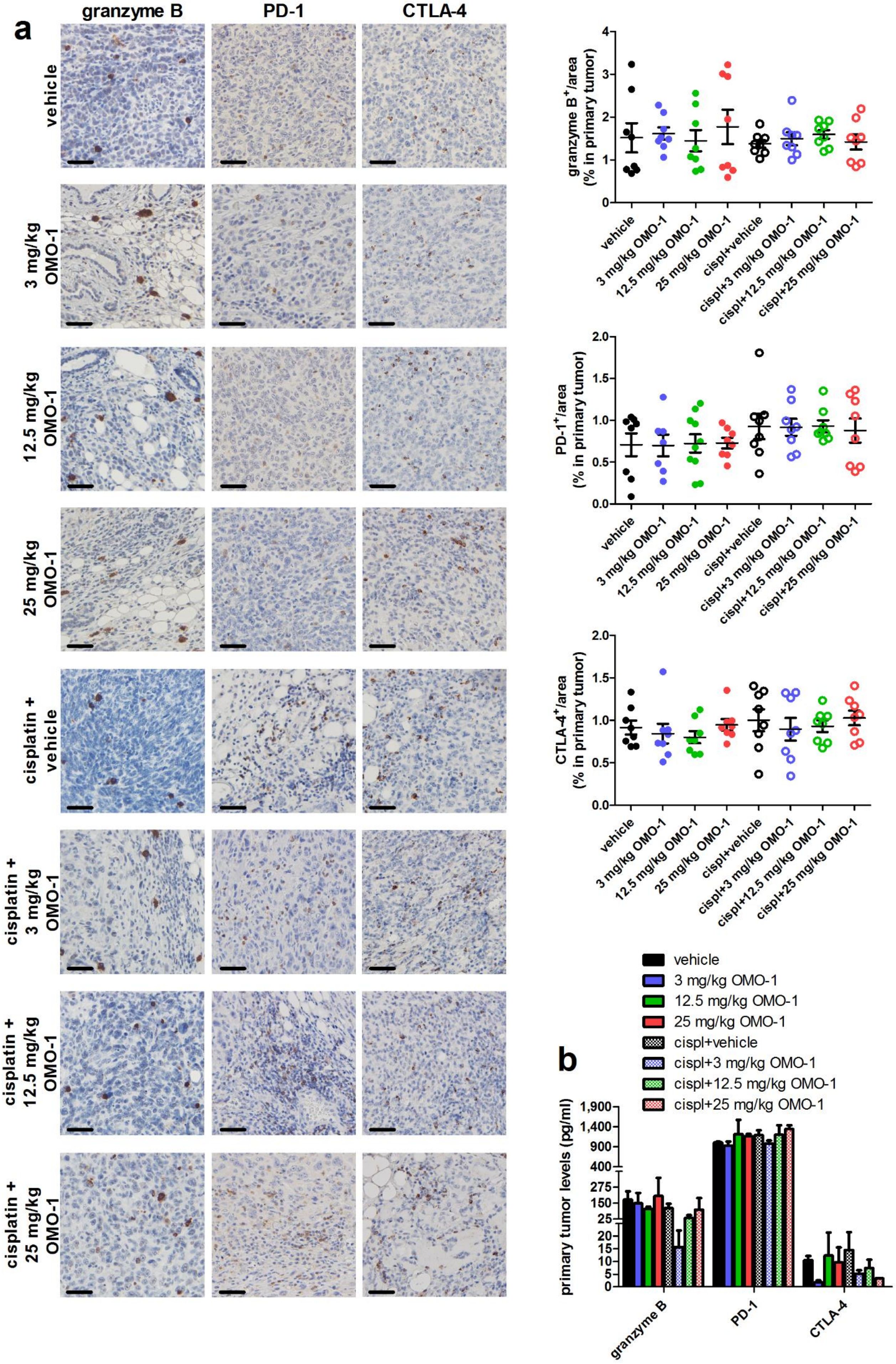

Supplementary Fig. 9. Tumor granzyme B, PD-1 and CTLA-4 upon OMO-1, cisplatin and combination treatment in a 4T1-based intraductal model.

(**a**) Immunohistochemistry for the activated cytotoxic T-cell marker granzyme B and immune checkpoint proteins PD-1 and CTLA-4 on paraffin sections of primary tumors from the different treatment groups at 39 d p.i. (n = 8; 2 slides per treatment group with 4 images per slide). Scale bars = 50 µm. (**b**) Levels of granzyme B, PD-1 and CTLA-4 in primary tumor lysates of the different treatment groups at 39 d p.i. (n = 5 for vehicle and cisplatin + vehicle, n = 4 for 25 mg/kg OMO-1 and cisplatin + 12.5 mg/kg OMO-1, n = 6 for all other treatment groups). Data are presented as the means +/- SEM.

Supplementary Fig. 10

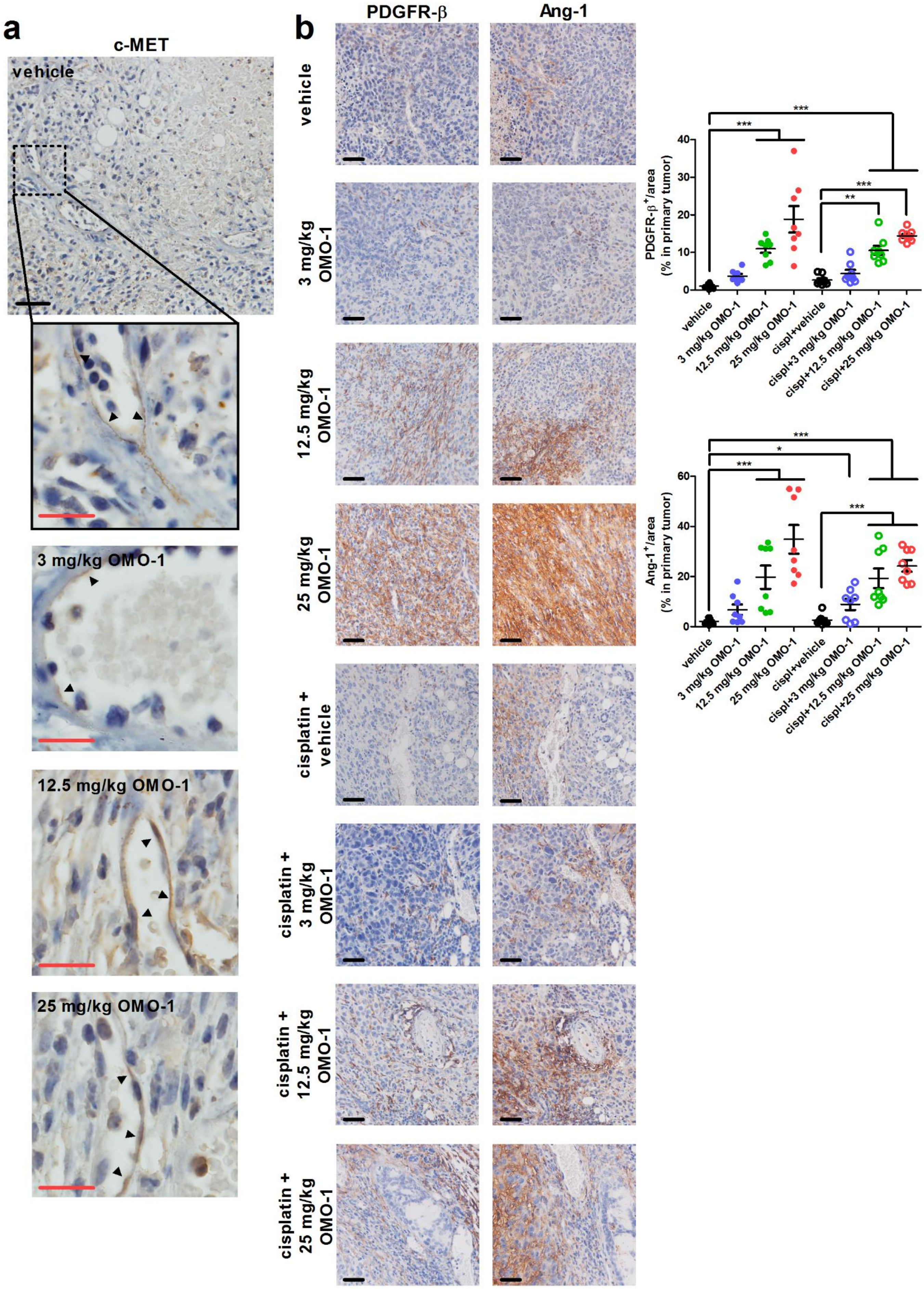

Supplementary Fig. 10. Vascular expression of c-MET and increased staining for additional pericyte markers in primary tumors of a 4T1-based intraductal model upon OMO-1 treatment.

(**a**) Immunohistochemistry for c-MET in the vascular bed of primary tumors treated with vehicle and increasing doses of OMO-1. Note that the majority of the primary tumor tissue does not show c-MET expression. (**b**) Immunohistochemistry for the additional pericyte markers PDGFR-β and Ang-1 on paraffin sections of primary tumors from the different treatment groups at 39 d p.i. ($n = 8$; 2 slides per treatment group with 4 images per slide). Black scale bars = 50 µm. Red scale bars = 20 µm. Data in panel **b** are presented as the means +/- SEM. *: $P < 0.05$, **: $P < 0.01$, ***: $P < 0.001$.

Supplementary Fig. 11

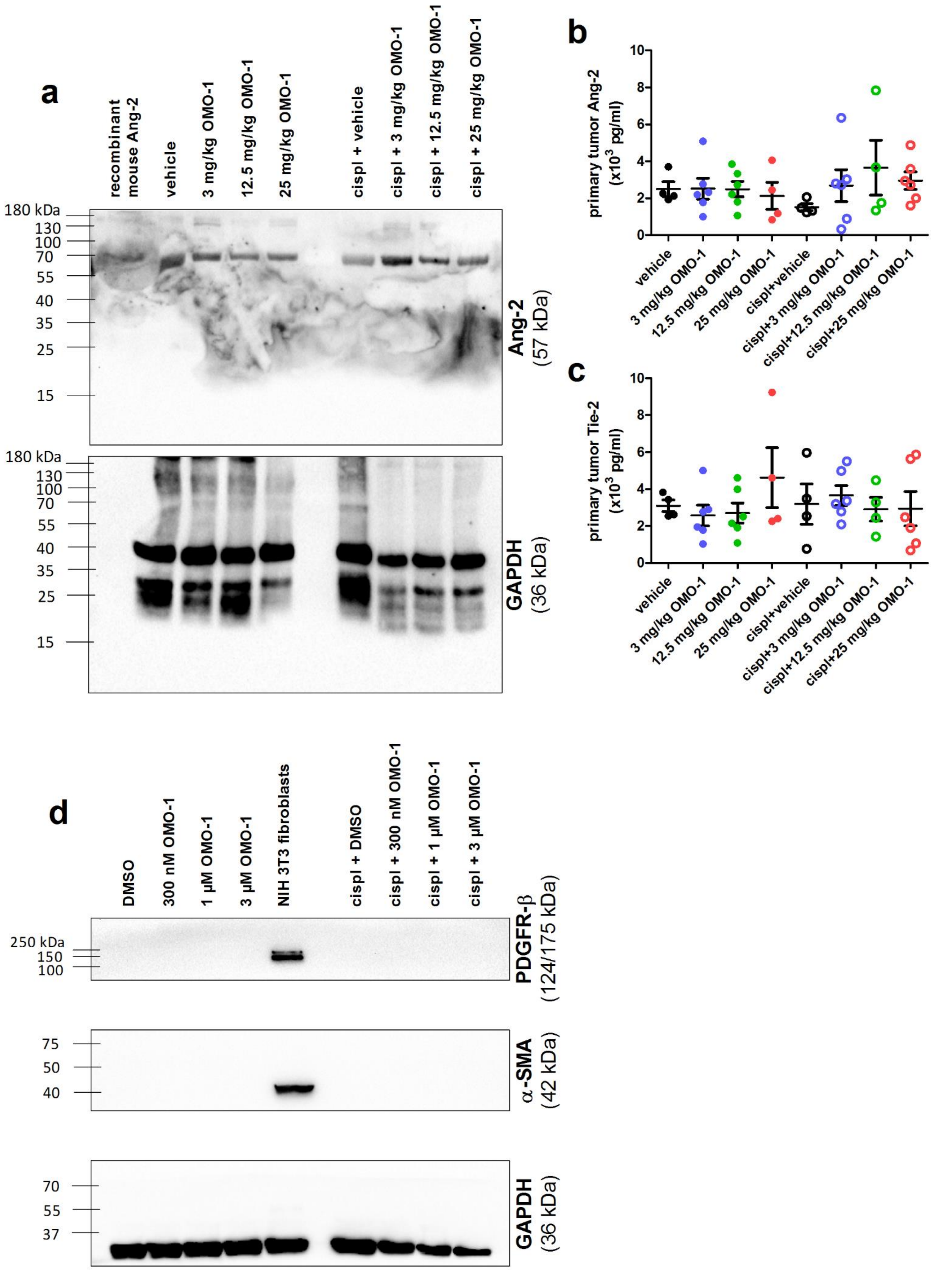

Supplementary Fig. 11. Ang-2 and Tie-2 levels *in vivo* and pericyte marker expression in cultured 4T1 cells upon OMO-1, cisplatin and combination treatment.

(**a**) Western blot for Ang-2 and GAPDH (loading control) in lysates of primary tumors from the different treatment groups at 39 d p.i. (**b**) Ang-2 levels in primary tumors from the different treatment groups at 39 d p.i. (n = 4 for vehicle, 25 mg/kg OMO-1, cisplatin + vehicle and cisplatin + 12.5 mg/kg OMO-1, n = 6 for all other treatment groups). (**c**) Tie-2 levels in primary tumors from the different treatment groups at 39 d p.i. (n = 4 for vehicle, 25 mg/kg OMO-1, cisplatin + vehicle and cisplatin + 12.5 mg/kg OMO-1, n = 6 for all other treatment groups). (**d**) Western blot of PDGFR-β and α-SMA expression in lysates of *in vitro* cultured 4T1 tumor cells following 48 h treatment with DMSO, OMO-1, cisplatin + DMSO and cisplatin + OMO-1. Lysates of NIH 3T3 fibroblasts were used as a positive control for PDGFR-β and α-SMA expression. Blots include samples of the same experiment. Data in panel **b** and **c** are presented as the means +/- SEM. *: $P < 0.05$.

Supplementary Fig. 12

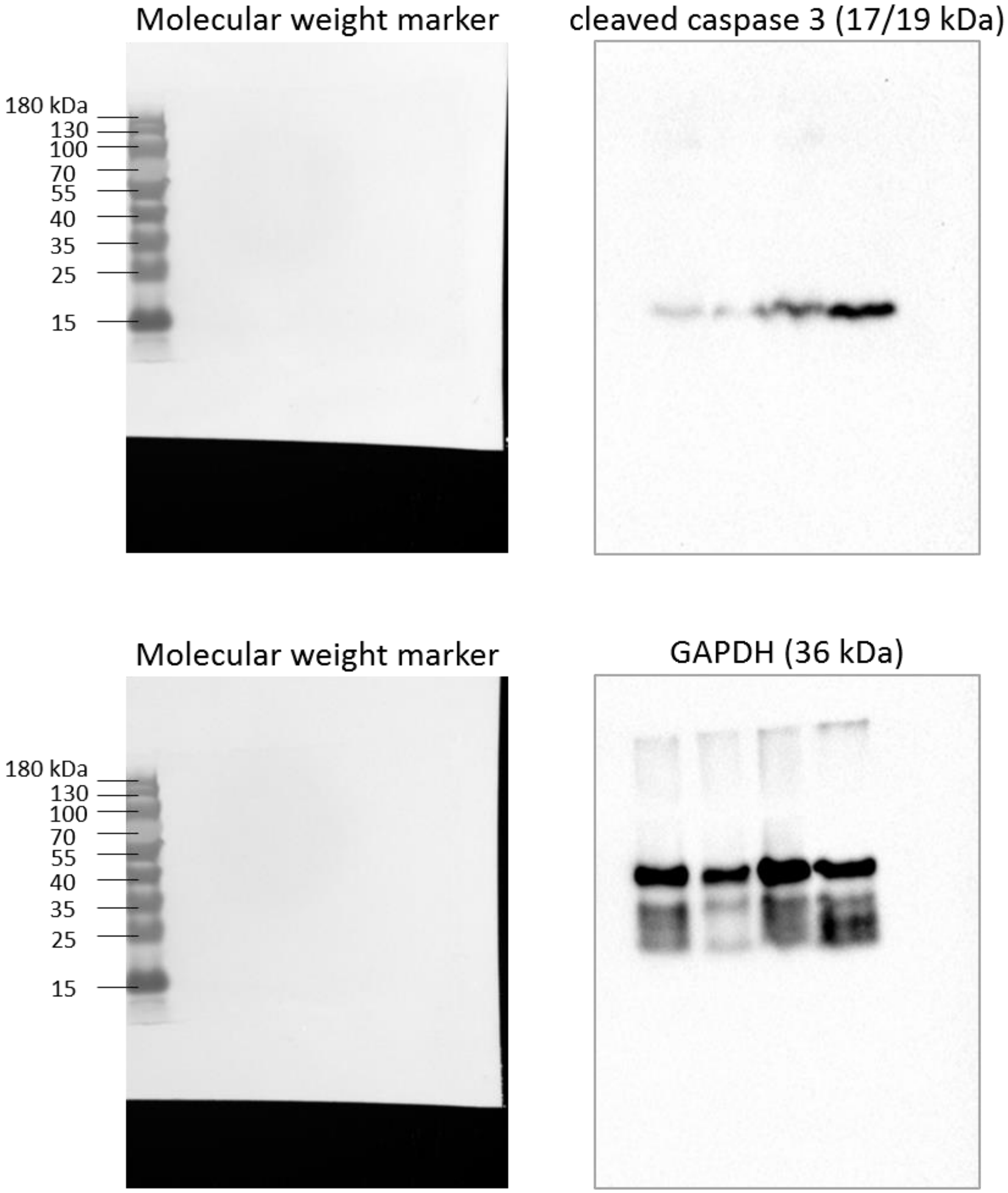

Supplementary Fig. 12. Full western blot images and molecular weight markers related to Fig. 5b.

Full western blot images and corresponding molecular weight markers for cleaved caspase 3 and GAPDH (loading control) in primary tumor lysates as shown in Fig. 5b.

Supplementary Fig. 13

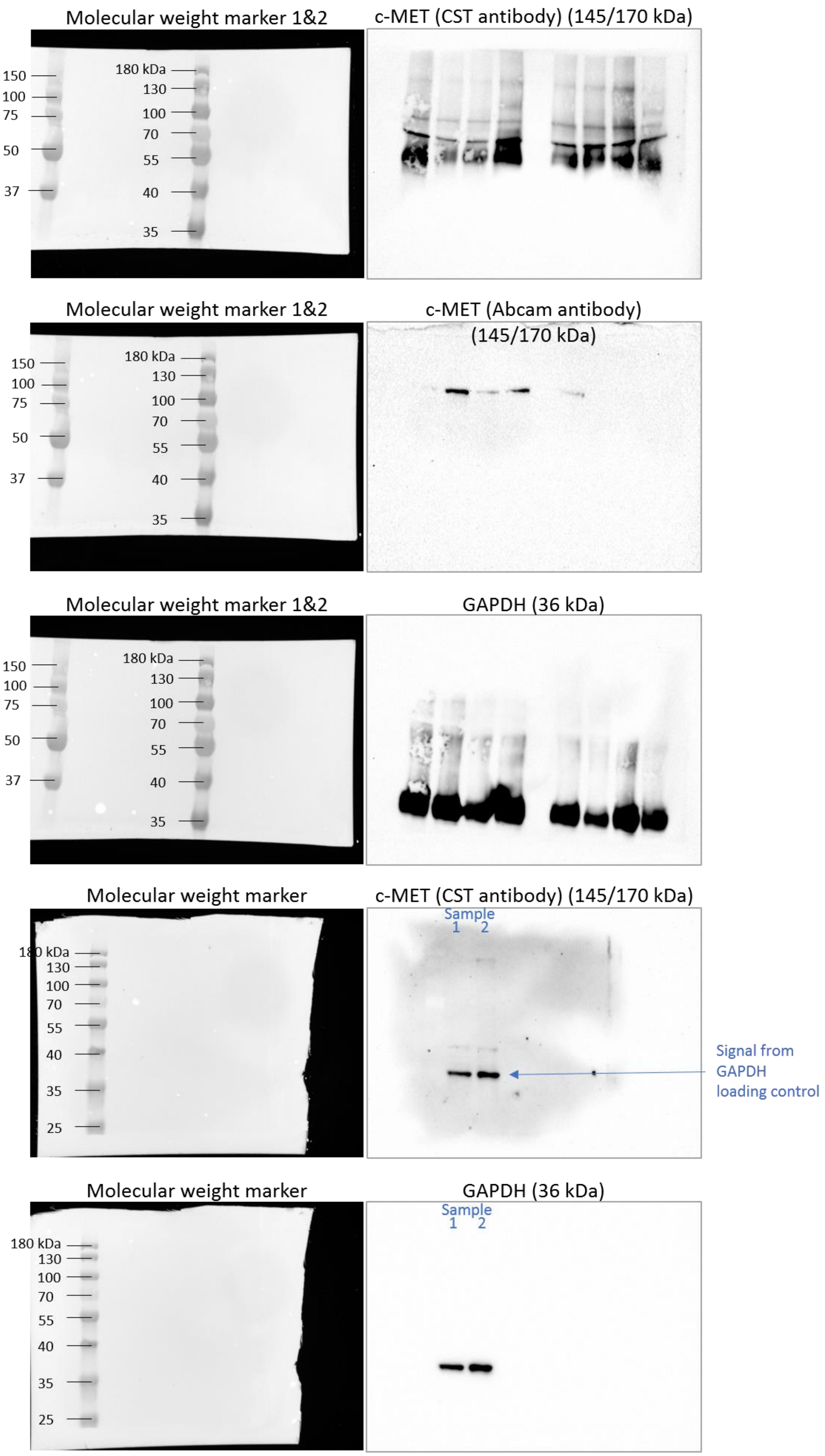

Supplementary Fig. 13. Full western blot images and molecular weight markers related to Supplementary Fig. 1b.

Full western blot images and corresponding molecular weight markers for c-MET (CST and Abcam antibody) and GAPDH (loading control) in primary tumor lysates and lysates of cultured 4T1 cells as shown in Supplementary Fig. 1b. Of note, only sample 2 on the western blots with cultured 4T1 cells is shown in Supplementary Fig. 1b.

Supplementary Fig. 14

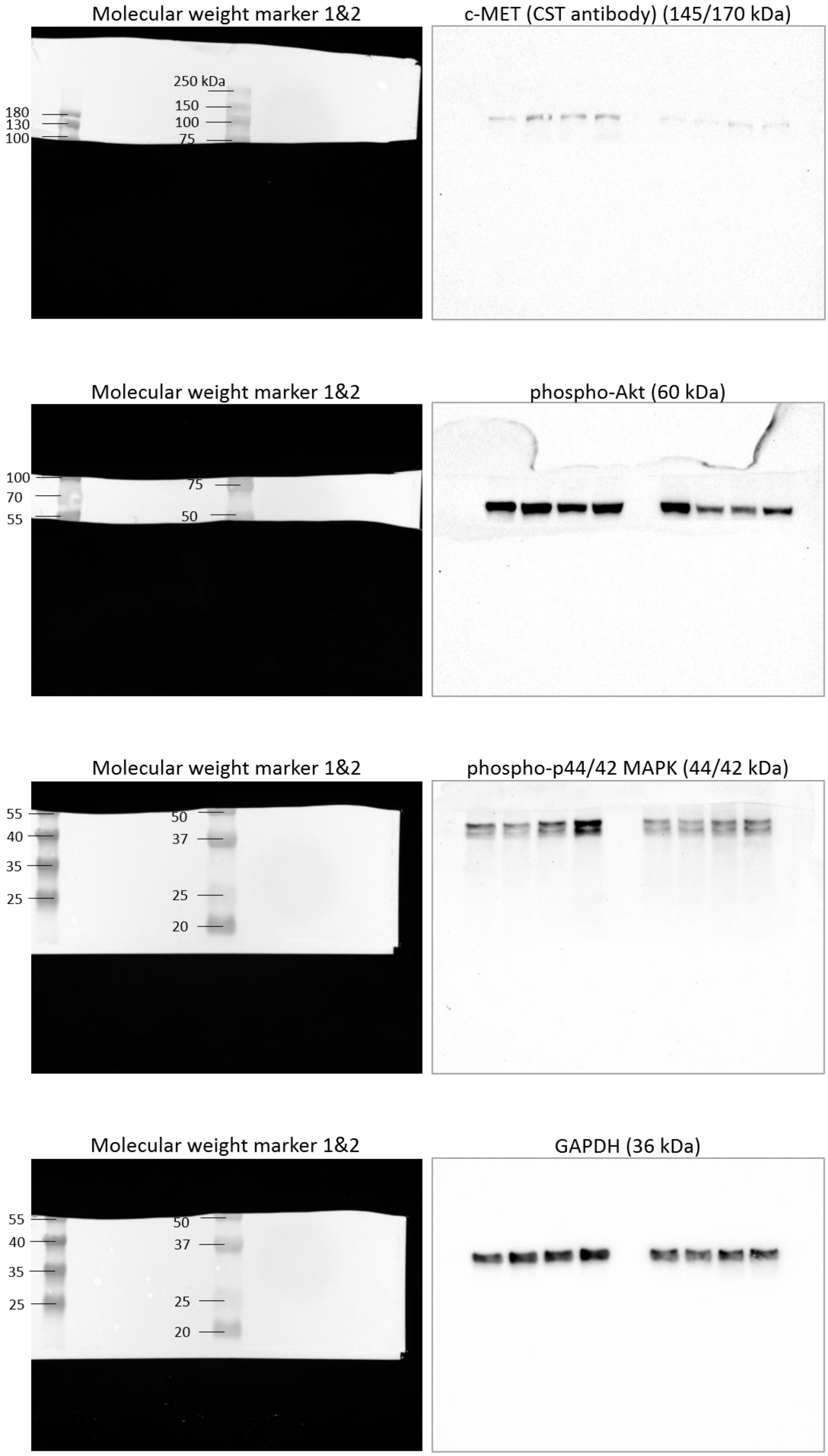

Supplementary Fig. 14. Full western blot images and molecular weight markers related to Supplementary Fig. 2a.

Full western blot images and corresponding molecular weight markers for c-MET, phospho-Akt, phospho-p44/42 MAPK and GAPDH (loading control) in lysates of cultured and treated 4T1 cells as shown in Supplementary Fig. 2a.

Supplementary Fig. 15

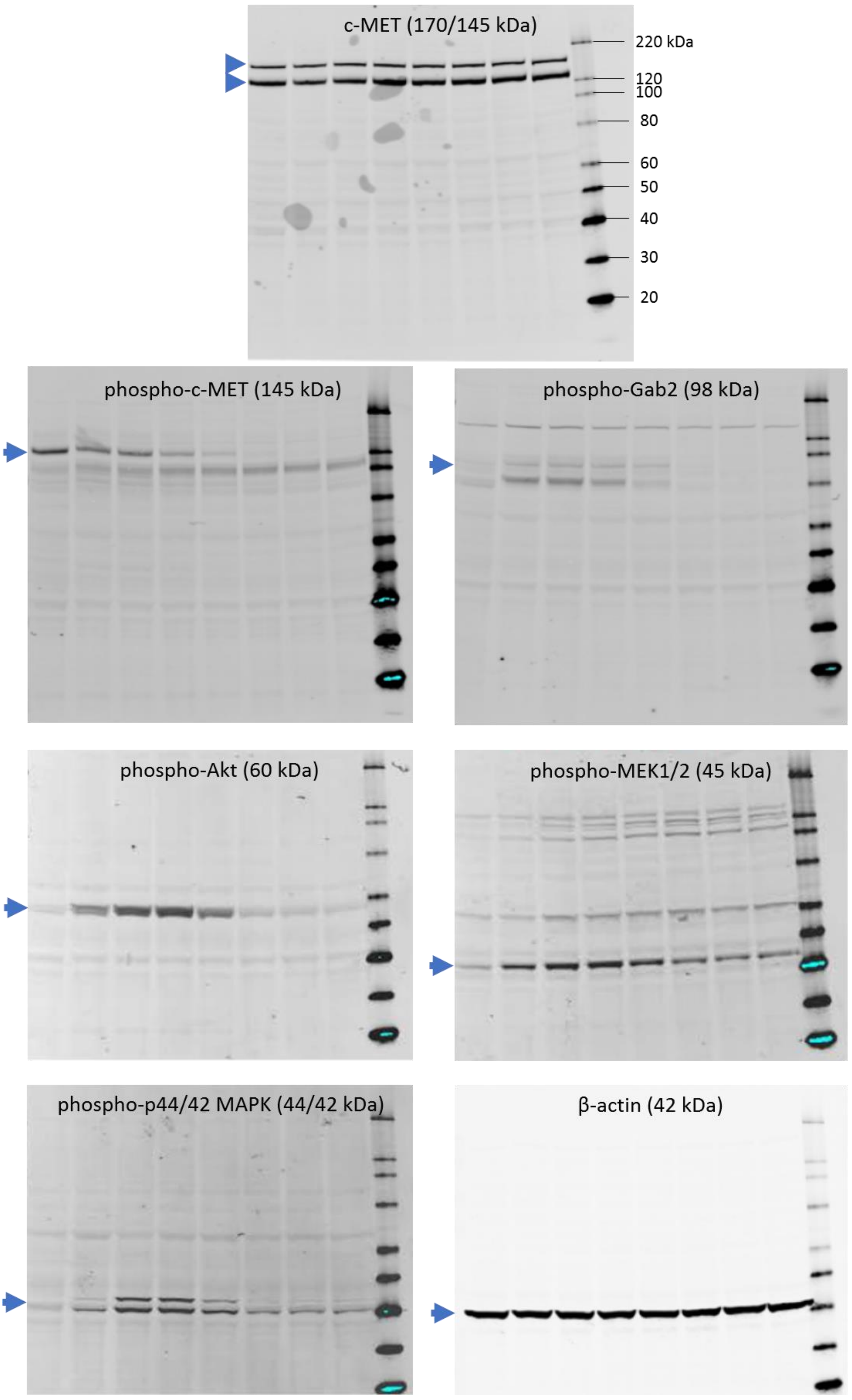

Supplementary Fig. 15. Full western blot images and molecular weight markers related to Supplementary Fig. 2b.

Full western blot images and corresponding molecular weight markers for c-MET, phospho-c-MET, phospho-Gab2, phospho-Akt, phospho-MEK1/2, phospho-p44/42 MAPK and β-actin (loading control) in lysates of cultured and treated NCI-H441 cells as shown in Supplementary Fig. 2b.

Supplementary Fig. 16

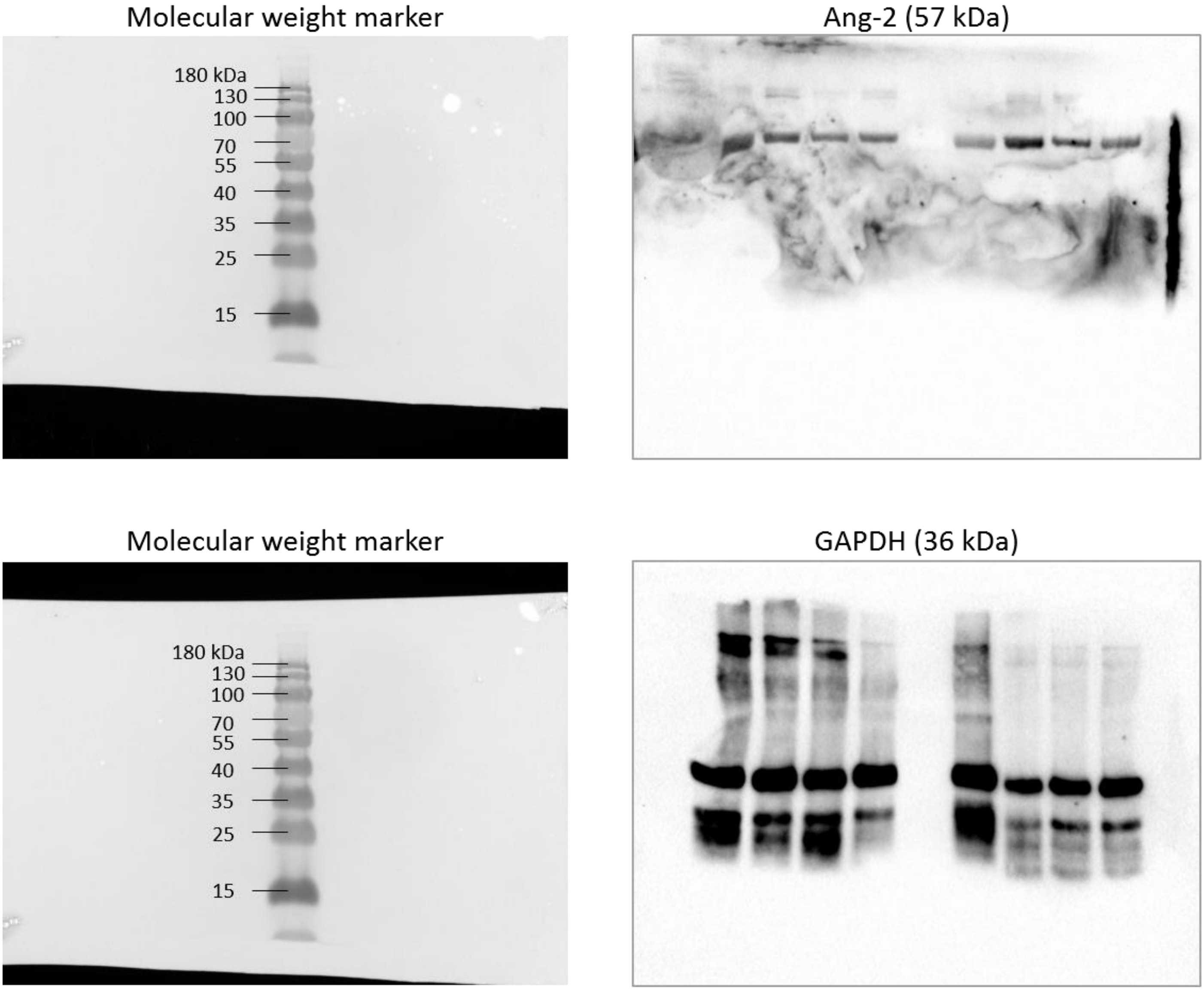

Supplementary Fig. 16. Full western blot images and molecular weight markers related to Supplementary Fig. 11a.

Full western blot images and corresponding molecular weight markers for Ang-2 and GAPDH (loading control) in primary tumor lysates as shown in Supplementary Fig. 11a.

Supplementary Fig. 17

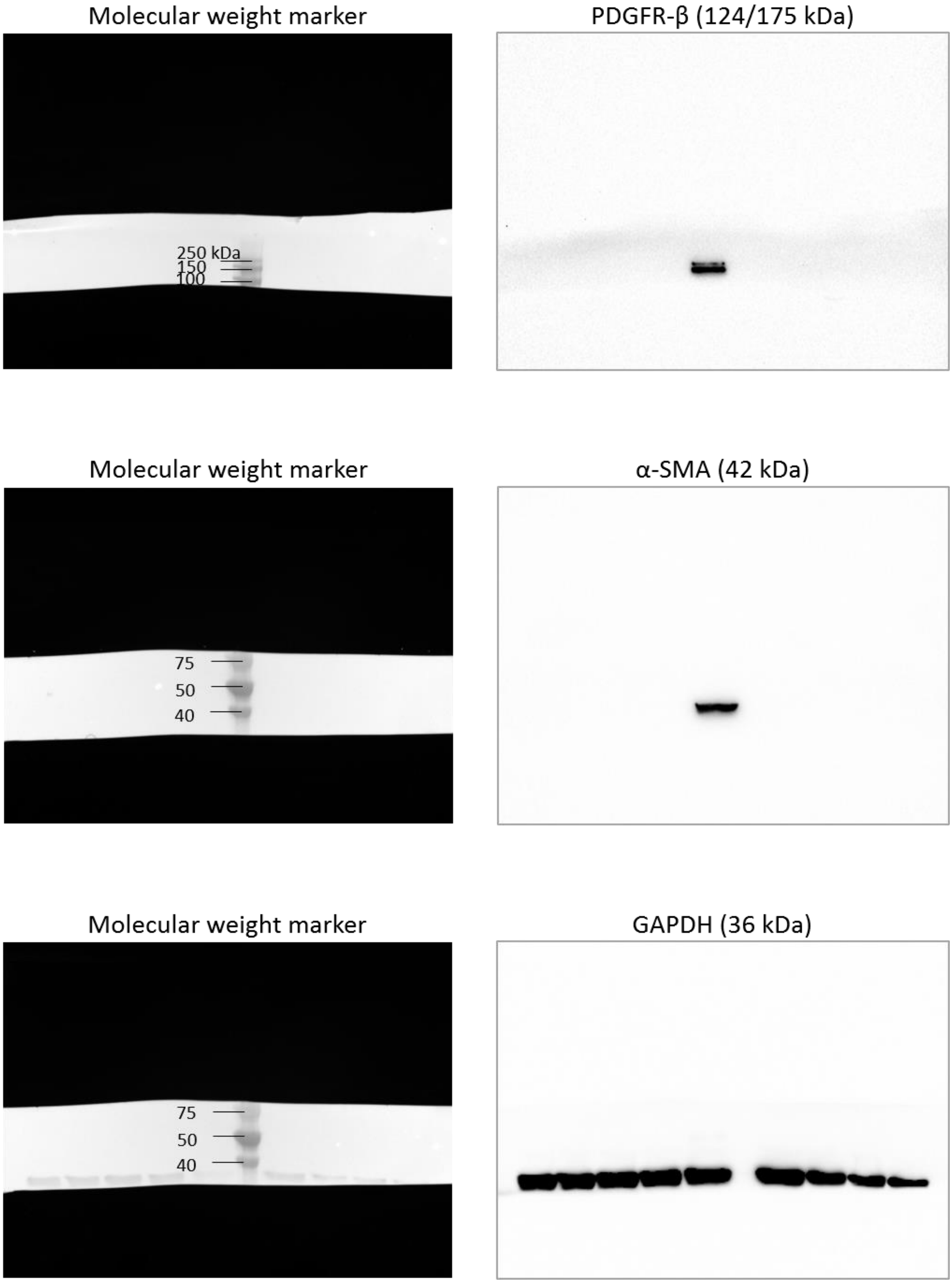

Supplementary Fig. 17. Full western blot images and molecular weight markers related to Supplementary Fig. 11d.

Full western blot images and corresponding molecular weight markers for PDGFR-β, α-SMA and GAPDH (loading control) in lysates of cultured and treated 4T1 cells as shown in Supplementary Fig. 11d.
