# Supplementary material for: OMO-1 reduces progression and enhances cisplatin efficacy in a 4T1-based non-c-MET addicted intraductal mouse model for triple-negative breast cancer
Source: NPJ Breast Cancer. 2021 Mar 17;7:27. doi: 10.1038/s41523-021-00234-8 (PMC7969607; doi:10.1038/s41523-021-00234-8)
Supplement: Supplementary file 2 — Reporting Summary Checklist [file 41523_2021_234_MOESM2_ESM.pdf]

nature research

Corresponding author(s): Jonas Steenbrugge

Last updated by author(s): Jan 18, 2021

Reporting Summary

Nature Research wishes to improve the reproducibility of the work that we publish. This form provides structure for consistency and transparency in reporting. For further information on Nature Research policies, see our Editorial Policies and the Editorial Policy Checklist.

Statistics

For all statistical analyses, confirm that the following items are present in the figure legend, table legend, main text, or Methods section.

n/a	Confirmed	
☐	☒	The exact sample size (*n*) for each experimental group/condition, given as a discrete number and unit of measurement
☐	☒	A statement on whether measurements were taken from distinct samples or whether the same sample was measured repeatedly
☐	☒	The statistical test(s) used AND whether they are one- or two-sided<br>*Only common tests should be described solely by name; describe more complex techniques in the Methods section.*
☐	☒	A description of all covariates tested
☐	☒	A description of any assumptions or corrections, such as tests of normality and adjustment for multiple comparisons
☐	☒	A full description of the statistical parameters including central tendency (e.g. means) or other basic estimates (e.g. regression coefficient) AND variation (e.g. standard deviation) or associated estimates of uncertainty (e.g. confidence intervals)
☐	☒	For null hypothesis testing, the test statistic (e.g. *F*, *t*, *r*) with confidence intervals, effect sizes, degrees of freedom and *P* value noted<br>*Give P values as exact values whenever suitable.*
☒	☐	For Bayesian analysis, information on the choice of priors and Markov chain Monte Carlo settings
☒	☐	For hierarchical and complex designs, identification of the appropriate level for tests and full reporting of outcomes
☒	☐	Estimates of effect sizes (e.g. Cohen's *d*, Pearson's *r*), indicating how they were calculated

Our web collection on statistics for biologists contains articles on many of the points above.

Software and code

Policy information about availability of computer code

Data collection	n/a
Data analysis	ImageJ and Graphpad Prism

For manuscripts utilizing custom algorithms or software that are central to the research but not yet described in published literature, software must be made available to editors and reviewers. We strongly encourage code deposition in a community repository (e.g. GitHub). See the Nature Research guidelines for submitting code & software for further information.

Data

Policy information about availability of data

All manuscripts must include a data availability statement. This statement should provide the following information, where applicable:

- Accession codes, unique identifiers, or web links for publicly available datasets
- A list of figures that have associated raw data
- A description of any restrictions on data availability

The datasets generated during and/or analysed during the current study are available from the corresponding author on reasonable request.

Field-specific reporting

Please select the one below that is the best fit for your research. If you are not sure, read the appropriate sections before making your selection.

☒ Life sciences ☐ Behavioural & social sciences ☐ Ecological, evolutionary & environmental sciences

For a reference copy of the document with all sections, see nature.com/documents/nr-reporting-summary-flat.pdf

Life sciences study design

All studies must disclose on these points even when the disclosure is negative.

Sample size	Due to mice sample variability, 4 mice were enrolled according to previous experiments.
Data exclusions	No data was exluded
Replication	All in vitro experiments, western blottings and IHC stainings were replicated at least twice.
Randomization	Mice were randomized into control or treatment groups.
Blinding	Researchers were not blinded

Reporting for specific materials, systems and methods

We require information from authors about some types of materials, experimental systems and methods used in many studies. Here, indicate whether each material, system or method listed is relevant to your study. If you are not sure if a list item applies to your research, read the appropriate section before selecting a response.

Materials & experimental systems

n/a	Involved in the study	
☐	☒	Antibodies
☐	☒	Eukaryotic cell lines
☒	☐	Palaeontology and archaeology
☐	☒	Animals and other organisms
☒	☐	Human research participants
☒	☐	Clinical data
☒	☐	Dual use research of concern

Methods

n/a	Involved in the study	
☒	☐	ChIP-seq
☒	☐	Flow cytometry
☒	☐	MRI-based neuroimaging

Antibodies

Antibodies used	Immunohisto/cytochemistry: anti-Ki67 (1:50, clone SP6; Thermo Fisher Scientific), anti-pan-cytokeratin (1:100, polyclonal, Abcam), anti-CD11b (1:4000, clone EPR1344, Abcam), anti-CD163 (1:500, clone EPR19518, Abcam), anti-Ly6G (1:1000; clone 1A8; BioLegend), anti-CD8a (1:50, clone 4SM15, Thermo Fisher Scientific), anti-granzyme B (1:1000, polyclonal, Abcam), anti-PD-1 (1:1000, clone EPR20665, Abcam), anti-CTLA-4 (1:500, clone CAL49, Abcam), FITC-conjugated anti-pimonidazole (1:25; clone 4.3.11.3; Hypoxyprobe), anti-CAIX (1:1000; clone NB100–417; Novus Biologicals), anti-CD31 (1:2000; clone EPR17259; Abcam), anti-CD31 (1:20, clone SZ31, Dianova), anti-α-SMA (1:2000, clone EPR5368, Abcam), anti-PDGFR-β (1:500, clone Y92, Abcam), anti-Ang-1 (1:500, polyclonal, Abcam), anti-cisplatin DNA-adducts (1:50, clone ICR4, Sigma-Aldrich), anti-cleaved caspase 3 (1:1000, clone 5A1E, Cell Signaling Technology).<br>Western blotting: anti-c-MET (1:1000, clone 25H2, Cell Signaling Technology), anti-c-MET (1:1000, clone EP1454Y, Abcam), anti-phospho-c-MET (Tyr1230/1234/1235; 1:1000, polyclonal, Thermo Fisher Scientific), anti-phospho-Gab2 (Tyr452; 1:1000, polyclonal, Cell Signaling Technology), anti-phospho-Akt (Ser473; 1:2000, clone D9E, Cell Signaling Technology), anti-phospho-Akt (1:1000, polyclonal, Cell Signaling Technology), anti-phospho-MEK1/2 (Ser217/221, 1:1000, polyclonal, Cell Signaling Technology), anti-phospho-p44/42 MAPK (Erk1/2; Thr202/Tyr204; 1:2000, clone D13.14.4E, Cell Signaling Technology), anti-phospho-p44/42 MAPK (Erk1/2; Thr202/Tyr204; 1:1000, clone E10, Cell Signaling Technology), anti-β-actin (1:5000, clone AC-15, Sigma-Aldrich), anti-Ang-2 (1:500, polyclonal, Novus Biologicals), anti-cleaved caspase 3 (1:1000, clone 5A1E, Cell Signaling Technology), anti-GAPDH (1:5000, clone EPR16891, HRP-conjugated, Abcam).
Validation	Validation was provided by the supplier companies.

Eukaryotic cell lines

Policy information about cell lines

Cell line source(s)	4T1-luc mammary tumor cells were a kind gift from Prof. Clare Isacke (Breakthrough Breast Cancer Research Centre, London, UK).
Authentication	The cell line was not authenticated.
Mycoplasma contamination	Cell cultures tested negative for mycoplasma contamination using a mycoplasma detection kit (PlasmoTestTM; Invivogen).
Commonly misidentified lines (See ICLAC register)	n/a

Animals and other organisms

Policy information about studies involving animals; ARRIVE guidelines recommended for reporting animal research

Laboratory animals	BALB/c mice were obtained from Envigo, Horst, The Netherlands.
Wild animals	n/a
Field-collected samples	n/a
Ethics oversight	Animal experiments were conducted according to Good Scientific Practice-principles and approved by the Ethical Committee (EC) of the Faculty of Veterinary Medicine, Ghent University (EC 2018-37 and amendment EC 2018-65).

Note that full information on the approval of the study protocol must also be provided in the manuscript.
